# DNMTs Play an Important Role in Maintaining the Pluripotency of Leukemia Inhibitory Factor-Dependent Embryonic Stem Cells

**DOI:** 10.1016/j.stemcr.2021.01.017

**Published:** 2021-02-25

**Authors:** Baojiang Wu, Yunxia Li, Bojiang Li, Baojing Zhang, Yanqiu Wang, Lin Li, Junpeng Gao, Yuting Fu, Shudong Li, Chen Chen, M. Azim Surani, Fuchou Tang, Xihe Li, Siqin Bao

**Affiliations:** 1 The State Key Laboratory of Reproductive Regulation and Breeding of Grassland Livestock, Inner Mongolia University, Hohhot 010020, China; 2 Research Center for Animal Genetic Resources of Mongolia Plateau, College of Life Sciences, Inner Mongolia University, Hohhot 010020, China; 3 College of Animal Science and Veterinary Medicine, Shenyang Agricultural University, Shenyang 110866, China; 4 Guangdong Provincial Key Laboratory of Proteomics, Department of Pathophysiology, School of Basic Medical Sciences, Southern Medical University, Guangzhou 510515, China; 5 Beijing Advanced Innovation Center for Genomics and Biomedical Pioneering Innovation Center, College of Life Sciences, Peking University, Beijing 100871, China; 6 Cancer Research UK and Medical Research Council Oxford Institute for Radiation Oncology, Department of Oncology, University of Oxford, Oxford OX3 7DQ, UK; 7 Wellcome Trust Cancer Research UK Gurdon Institute, Tennis Court Road, University of Cambridge, Cambridge CB2 1QN, UK; 8 Peking–Tsinghua Center for Life Sciences, Peking University, Beijing 100871, China; 9 Ministry of Education Key Laboratory of Cell Proliferation and Differentiation, Beijing 100871, China; 10 Inner Mongolia Saikexing Institute of Breeding and Reproductive Biotechnology in Domestic Animals, Huhhot 011517, China

**Keywords:** embryonic stem cells, leukemia inhibitory factor, epigenetic, genomic imprinting, DNA methylation, DNMTs, pluripotency, mouse, self-renew, differentiation

## Abstract

Naive pluripotency can be maintained in medium with two inhibitors plus leukemia inhibitory factor (2i/LIF) supplementation, which primarily affects canonical WNT, FGF/ERK, and JAK/STAT3 signaling. However, whether one of these three supplements alone is sufficient to maintain naive self-renewal remains unclear. Here we show that LIF alone in medium is sufficient for adaptation of 2i/L-ESCs to embryonic stem cells (ESCs) in a hypermethylated state (L-ESCs). Global transcriptomic analysis shows that L-ESCs are close to 2i/L-ESCs and in a stable state between naive and primed pluripotency. Notably, our results demonstrate that DNA methyltransferases (DNMTs) play an important role in LIF-dependent mouse ESC adaptation and self-renewal. LIF-dependent ESC adaptation efficiency is significantly increased in serum treatment and reduced in *Dnmt3a* or *Dnmt3l* knockout ESCs. Importantly, unlike epiblast stem cells, L-ESCs contribute to somatic tissues and germ cells in chimeras. L-ESCs cultured under such simple conditions as in this study would provide a more conducive platform to clarify the molecular mechanism of ESCs in *in vitro* culture.

## Highlights

• LIF alone supports ESC self-renewal and pluripotency in chemically defined media
• L-ESCs re-establish the epigenetic state in LIF adaptation
• DNMTs are important for LIF adaptation and L-ESC self-renewal
• L-ESCs contribute to somatic tissues and germ cells in chimeras

## Graphical Abstract

**Figure:**
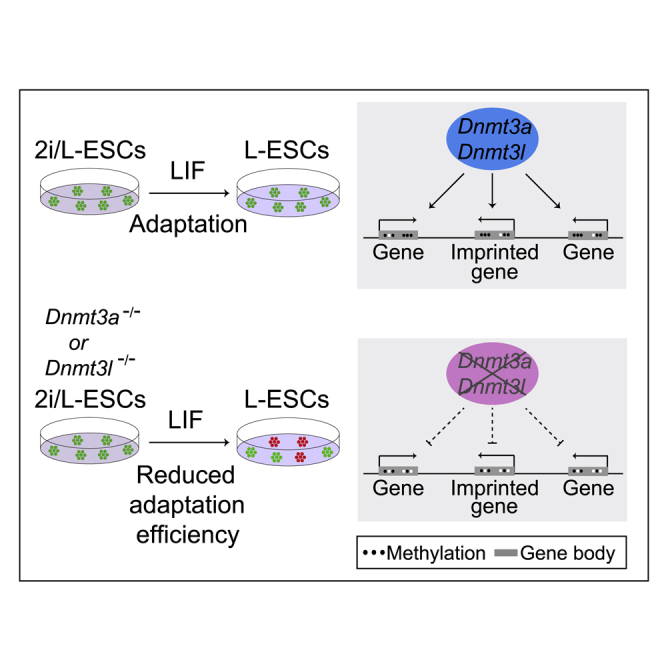

## Teaser

In this article, Bao and colleagues show that LIF alone supports ESC self-renewal and pluripotency in chemically defined media. L-ESCs exhibit distinct transcriptional features and re-establish DNA hypermethylation. Moreover, DNMTs are important for L-ESC adaptation and self-renewal. Notably, L-ESCs also contribute to somatic tissues and germ cells in chimeras.

## Introduction

Mouse embryonic stem cells (ESCs) are isolated from the inner cell mass (ICM) of the pre-implantation embryo (Martin, 1981). Since pluripotent mouse ESCs were first established 4 decades ago, various culture systems of ESCs have been developed, including initially using feeder/serum/cytokines, then leukemia inhibitory factor (LIF) and bone morphogenetic protein 4 (BMP4) (Smith et al., 1988; Williams et al., 1988; Ying et al., 2003), and more recently using 2i/LIF (two inhibitors, CHIR99021 and PD0325901, and LIF) (Ying et al., 2008). It is generally believed that the optimal culture conditions for ground-state ESCs comprise the three-additive 2i/LIF supplement, which affects canonical WNT, FGF/ERK, and JAK/STAT3 signals, respectively (Ohtsuka et al., 2015). It has been reported that the combination of any two of these three supplements was sufficient to maintain naive self-renewal of ESCs (Hackett et al., 2017).

LIF is the most pleiotropic member of the interleukin-6 family of cytokines and utilizes a receptor that consists of the LIF receptor and gp130 (Ohtsuka et al., 2015). LIF is able to activate three intracellular signaling pathway: the JAK/STAT pathway, the PI3K/AKT pathway, and the SH2 domain-containing tyrosine phosphatase/mitogen-activated protein kinase pathway. LIF has antagonistic effects in different cell types, including stimulating or inhibiting cell proliferation, differentiation, and survival. Since LIF was detected in an extract from feeder cells and has been used for most mouse ESC media, it has been fully demonstrated to be an important supplement for ESC self-renewal and pluripotency (Gao et al., 2019; Williams et al., 1988; Yang et al., 2017; Ying et al., 2003, 2008). Nevertheless, essential LIF/STAT3 functions can be compensated for by activation of canonical WNT signaling and inhibition of FGF/ERK in the established culture system for self-renewal of ESCs (Ohtsuka et al., 2015). However, the consequences LIF/STAT3 signaling alone and precise regulatory mechanisms for ESC self-renewal have remained largely elusive.

Mouse ESCs cultured under different culture conditions exhibit distinct DNA methylation patterns. The ESCs (2i/L-ESCs) cultured in 2i/LIF medium are globally DNA hypomethylated, whereas ESCs grown in classical medium containing feeders, serum, and LIF (S/L-ESCs) show global DNA hypermethylation (Leitch et al., 2013; Stadler et al., 2011). In addition, DNA methylation levels were shown to be reversible between S/L-ESCs and 2i/L-ESCs (Leitch et al., 2013). Previous research reported prolonged that MEK1/2 suppression impairs the epigenetic and genomic integrity as well as the developmental potential of ESCs, in part through the downregulation of DNA methylation (Choi et al., 2017; Yagi et al., 2017). In addition, DNA methylation plays an important role in embryonic development, stem cell differentiation, and cell fate conversion (Bourc'his et al., 2001; Cirio et al., 2008; Kaneda et al., 2004; Li et al., 1992; Webster et al., 2005). We also showed that hypermethylation is a key point for expanded pluripotency of ESCs in chemically defined medium (Bao et al., 2018; Wu et al., 2020).

The combination of 2i supports the self-renewal of ESCs in serum-free culture without LIF; however, addition of LIF in 2i culture condition further promotes self-renewal of ESCs, suggesting the synergistic effect of 2i and LIF (Ying et al., 2008). PD0325901 suppresses the differentiation of ESCs but does not support proliferation (Huang et al., 2015; Ying et al., 2008). CHIR99021 is highly specific to GSK3 and it alone is not sufficient to support the self-renewal of ESCs in serum-free culture (Ying et al., 2008). In this study, we focus on JAK/STAT3 signaling and show that LIF alone in serum-free and 2i-free medium is able to support ESC self-renewal and pluripotency as well as developmental potency. Our data also indicate that DNA methyltransferases (DNMTs) play an important role in LIF-dependent mouse ESC adaptation and self-renewal. The detailed analysis of LIF-alone-dependent mouse ESCs provides new insight into global DNA (de)methylation and also provides a rich resource for future studies on ESCs in *in vitro* culture.

## Results

### LIF Alone Supports ESC Self-Renewal and Pluripotency in Chemically Defined Media

**Figure 1:**
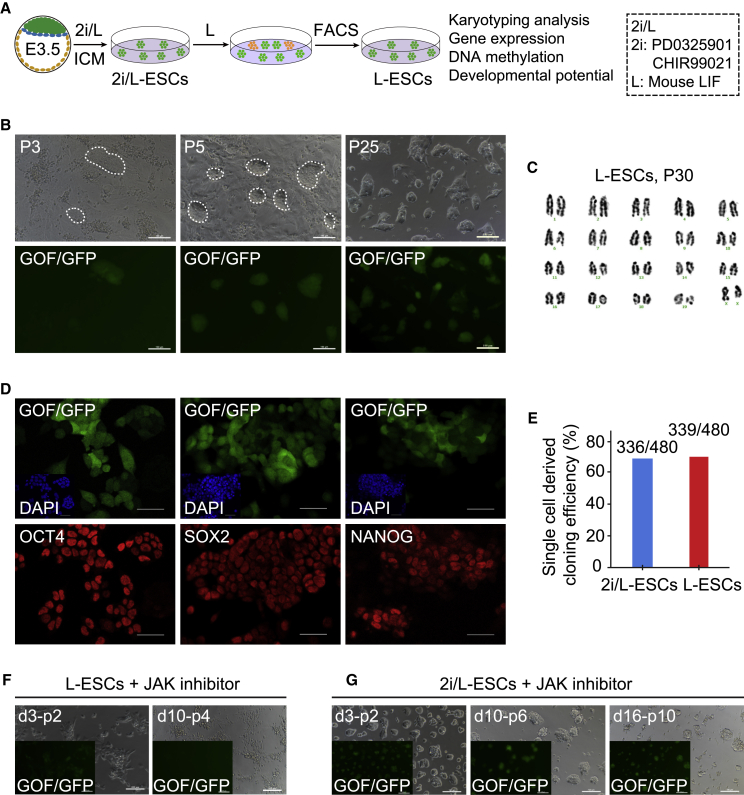
LIF Alone Supports ESC Self-Renewal and Pluripotency (A) Experimental outline of the L-ESC derivation procedure from 2i/L-ESCs. (B) 2i/L-ESCs were switched to L medium and cultured to passages 3 (P3), P5, and P25. Here we used 2i/L-ESCs with the GOF/GFP reporter. Scale bars, 100 μm. See also Figure S1A. (C) Karyotyping of L-ESCs (P30, n = 50, results of three independent experiments). (D) Immunostaining of OCT4, SOX2, and NANOG in L-ESCs (results of three independent experiments). Scale bars, 50 μm. (E) Single-cell clonogenicity efficiency in L-ESCs and 2i/L-ESCs (n = 480 single cells of L-ESCs and 2i/L-ESCs, respectively; results of three independent experiments). (F) L-ESCs were treated with JAK inhibitor I after day 3 P2 and day 10 P4 (results of three independent experiments). Scale bars, 100 μm. (G) 2i/L-ESCs were treated with JAK inhibitor I after day 3 P2, day 10 P6, and day 16 P10 (results of three independent experiments). Scale bars, 100 μm.

Serum plus LIF (S/L) medium and 2i/LIF medium (based on N2B27) are two typical ESC culture media. In particular, LIF was found in almost all mouse ESC culture media *in vitro*. Therefore, we sought to determine whether LIF alone is capable of driving continuous cycles of self-renewal of ESCs in serum-free and 2i-free medium. Here we used seven Oct4-ΔPE-GFP (GOF/GFP, mixed background of MF1, 129/sv, and C57BL/6J strains) × 129/sv F1 mouse (Yoshimizu et al., 1999) ESC lines (W1, W2, W4, W5, W6, SQ3.3, and X/GFP; the sex of the cell lines is indicated in Figure S1A), which were directly derived in 2i/LIF medium (passage [p] 15–p20) and then switched to chemically defined LIF (1000 IU/mL)-alone medium based on N2B27 (L medium) (Figures 1A and S1A). Initially ESCs showed signs of differentiation, such as flattening of colonies and reduction of GOF/GFP positivity for pluripotency-related transcription factor *Oct4* (Figure 1B). However, in p3–p5, some GOF/GFP^+^ colonies similar to those in undifferentiated ESCs were discovered in LIF-alone medium (Figure 1B). We designated these LIF-dependent GOF/GFP^+^ ESCs in chemically defined LIF-alone medium as L-ESCs. GOF/GFP^+^ colonies increased gradually with further passages. L-ESCs were successfully derived from all seven 2i/L-ESCs (Figures 1B and S1A).

Next, we performed fluorescence-activated cell sorting (FACS) on multiple L-ESC lines, and the sorted GOF/GFP^+^ L-ESCs were cultured in L medium. The percentage of GOF/GFP^+^ L-ESCs (p14–p42) ranged from 56% to 99% in several ESC lines (Figure S1B). After two or more repeated FACS assays for each L-ESC line (Figure S1B), GOF/GFP^+^ L-ESCs reached nearly 98% purity, which was similar to the control 2i/L-ESCs (Figure S1B). These results indicate that LIF alone can maintain FACS-purified GOF/GFP^+^ L-ESCs in an undifferentiated pluripotent state (Figures 1B and S1B), with stable growth over 40 passages (Figure S1C) and high alkaline phosphatase (AP) activity (Figure S1D). The established L-ESC lines have a normal karyotype (Figures 1C and S1E) and express pluripotent markers OCT4, SOX2, and NANOG, similar to 2i/L-ESCs, confirmed by immunofluorescence (Figures 1D and S1F). In mouse ESCs, the cells that exhibit some features of two-cell embryos comprise less than 1% (Li and Izpisua Belmonte, 2018; Macfarlan et al., 2012). Interestingly, L-ESCs also retained two-cell features, such as ZSCAN4 and MERVL activities, demonstrated by immunostaining (Figure S1G). In addition, it has been reported that both X chromosomes are active in female naive ESCs (Pasque et al., 2018; Schulz et al., 2014); concurrent with this, our immunostaining showed no H3K27me3 foci in female L-ESCs, suggesting that both X chromosomes are activated (Figure S1H). To further verify that the two X chromosomes in L-ESCs are active, we used the 2i/L-ESC line with X/GFP (X/GFP is expressed upon X chromosome reactivation) (Bao et al., 2009) to induce L-ESCs, and found that both X chromosomes in L-ESCs were activated (Figure S1I). These results suggest that L-ESCs possess some of the characteristics of 2i/L-ESCs.

For a further stringent test of the pluripotency of L-ESCs, we examined the ability for clone formation from the single-cell level. We observed that L-ESC clones could be derived from single cells in chemically defined LIF-alone medium with high efficiency, comparable to those from 2i/L-ESCs (Figure 1E). Furthermore, to examine how essential LIF is in maintaining L-ESCs, we withdrew LIF and then added JAK inhibitor I, and observed significantly impaired propagation of L-ESCs with rapid differentiation (Figure 1F). However, LIF withdrawal and JAK inhibitor I addition did not affect the self-renewal of 2i/L-ESCs until p10 (Figure 1G). Taken together, our results suggest that LIF is an important and essential regulator in the maintenance of L-ESCs. In contrast to the previous notion that LIF and 2i both play an important role in ESC self-renewal, and establish a unique ground state of ESCs, in this study we showed that LIF alone is capable of supporting ESCs for self-renewal and proliferation over p40.

### Global Transcriptional Features of L-ESCs

**Figure 2:**
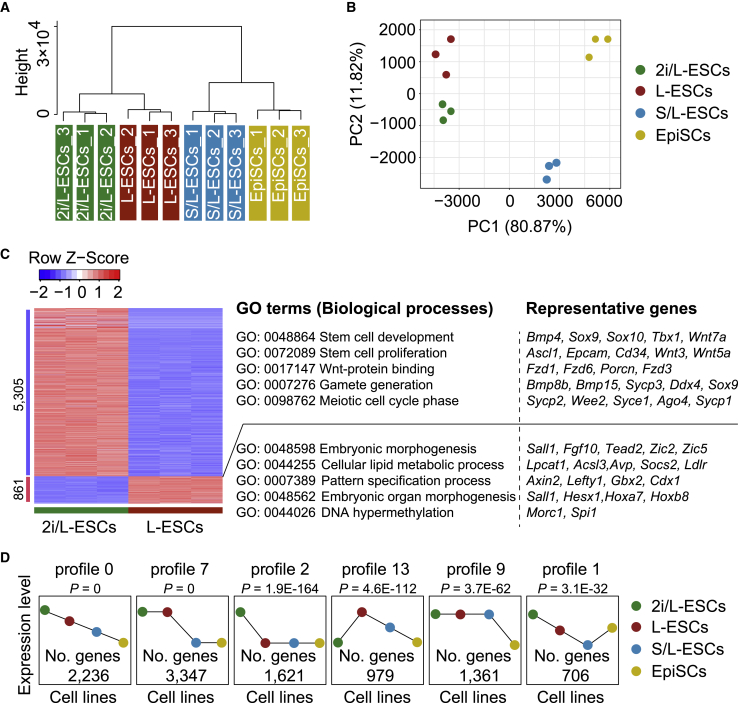
Analyses of Molecular Features of L-ESCs (A) Unsupervised hierarchical clustering of the transcriptome from three biological replicates (n = 3) of four pluripotent stem cell lines. (B) Principal component analysis of gene expression from the transcriptomes of three biological replicates (n = 3) of four pluripotent stem cells. (C) Heatmap showing differentially expressed genes (mean log2(normalized read counts) > 2, log2(fold change) > 2, adjusted p < 0.05) in L-ESCs (n = 3) compared with 2i/L-ESCs (n = 3). Significantly enriched gene ontology (GO) terms and representative genes in each cluster are listed on the right. (D) Comparison of L-ESCs, 2i/L-ESCs, S/L-ESCs, and EpiSCs. Among differentially expressed genes, a total of 3,347 genes (profile 7) were significantly highly expressed in L-ESCs and 2i/L-ESCs compared with S/L-ESCs and EpiSCs; a total of 1,621 genes (profile 2) were significantly upregulated in 2i/L-ESCs compared with L-ESCs, S/L-ESCs, and EpiSCs (n = 3 biological replicates of four pluripotent stem cell lines).

To examine whether L-ESCs have distinct molecular features, we carried out RNA sequencing (RNA-seq) on L-ESCs, 2i/L-ESCs, S/L-ESCs and epiblast stem cells (EpiSCs). Unsupervised hierarchical clustering and principal component analysis showed that L-ESCs were close to 2i/L-ESCs (Figures 2A and 2B) and appeared to be at an intermediate state between naive ESCs and primed EpiSCs (Figure 2A). Comparing L-ESCs and 2i/L-ESCs, L-ESC differentially expressed genes were related to embryonic morphogenesis, cellular lipid metabolic processes, pattern specification processes, embryonic organ morphogenesis, and DNA hypermethylation, whereas 2i/L-ESC differentially expressed genes were related to stem cell development, stem cell proliferation, gamete generation, and meiotic cell-cycle phase (Figure 2C). In addition, our data indicated that most general naive markers in L-ESCs were clustered with 2i/L-ESCs (Figures S2A and S2B), and primed markers in L-ESCs exhibit intermediate state between 2i/L-ESCs and EpiSCs (Figure S2C). Furthermore, β-CATENIN and ERK play central roles in balancing differentiation and self-renewal (Aulicino et al., 2020); hence, we tested the β-CATENIN and ERK levels in L-ESCs. Notably, some WNT signaling-related genes and the protein level of β-CATENIN were differentially expressed in L-ESCs compared with 2i/L-ESCs (Figures S2D and S2E). Meanwhile, the protein level of p-ERK was significantly upregulated in L-ESCs compared with 2i/L-ESCs (Figure S2E). These data show that L-ESCs display distinct molecular features for pluripotency. Meanwhile, we used the short time-series expression miner (STEM) method (Ernst and Bar-Joseph, 2006) to analyze gene expression profiles on L-ESCs, 2i/L-ESCs, S/L-ESCs, and EpiSCs. Interestingly, 3,347 differentially expressed genes (profile 7) were significantly highly expressed in L-ESCs and 2i/L-ESCs compared with S/L-ESCs and EpiSCs (Figure 2D). Notably, a total of 1,621 genes (profile 2) were significantly upregulated in 2i/L-ESCs compared with L-ESCs, S/L-ESCs and EpiSCs (Figure 2D). In addition, previous studies showed that 2i/L-ESCs were equal to the ICM of E3.5 blastocysts, whereas S/L-ESCs were close to the epiblast of E4.5–E5.5 (Boroviak et al., 2014). In our study, we found that 2i/L-ESCs and L-ESCs are closer than S/L-ESCs based on their molecular features as revealed by the RNA-seq analysis (Figure 2A). These RNA-seq analyses suggest that L-ESCs are in a stable state between naive and primed pluripotency. Recently, Austin Smith and colleagues (Kinoshita et al., 2020; Yu et al., 2020) described the intermediate formative stem cells (FS cells), and most FS cell-related genes were lower in L-ESCs compared with 2i/L-ESCs (Figure S2F). Therefore, L-ESCs may represent a distinct intermediate state of ESC *in vitro* culture; the relevant *in vivo* developmental stage remains to be further determined.

### L-ESCs Exhibit DNA Hypermethylation and Reserve Genomic Imprints

**Figure 3:**
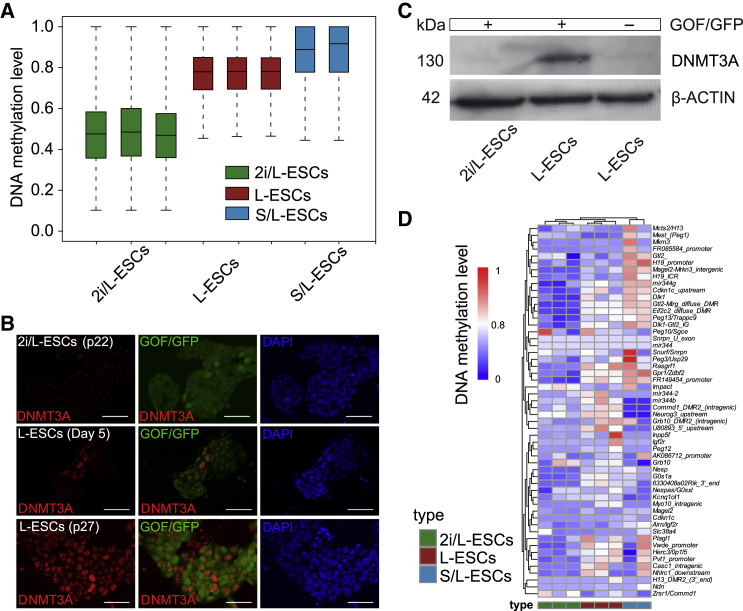
DNA Methylation Pattern of L-ESCs (A) DNA methylation level of 2-kb genomic tiles in L-ESCs (n = 3), 2i/L-ESCs (n = 3), and S/L-ESCs (n = 2). Source data are provided in Table S1. (B) Immunostaining of DNMT3A in 2i/L-ESCs and different passages of L-ESCs (results of three independent experiments). Scale bars, 50 μm. (C) Western blotting analysis for DNMT3A in early adaptation stage (day 5) GOF/GFP^+^ and GOF/GFP^−^ L-ESCs (results of three independent experiments). (D) Heatmap showing DNA methylation level of ICRs in L-ESCs (n = 3), 2i/L-ESCs (n = 3), and S/L-ESCs (n = 2).

ESCs cultured in 2i/LIF or LIF plus serum supplemented media represent two states of pluripotency of ESCs (Atlasi et al., 2019; Marks et al., 2012). Despite their similarities in pluripotency, 2i/LIF- and S/L-ESCs rely on different signaling pathways and display strong differences in transcriptional and epigenetic landscapes (Habibi et al., 2013; Joshi et al., 2015; Ter Huurne et al., 2017). Here, we asked whether there are different DNA methylation levels among L-ESCs, 2i/L-ESCs, and S/L-ESCs. Whole-genome bisulfite sequencing (WGBS) was performed, and DNA methylation profiling of L-ESCs was compared with 2i/L-ESCs and S/L-ESCs. To avoid the effect of sex on DNA methylation in L-ESCs, the male cell lines (2i/L-ESCs and L-ESCs) were selected for DNA methylation analysis. The levels of DNA methylation in L-ESCs (median CpG methylation of ∼80%) were comparable to those of S/L-ESCs (median ∼90%) and higher than those of 2i/L-ESCs (median ∼30%) (Figure 3A). This DNA methylation occurs across most methylated regions, including intragenic, intergenic, exon, intron, short and long interspersed nuclear elements, and long terminal repeats (Figure S3A). In addition, expression of DNA methylation-associated genes were assessed using qPCR. As expected, DNMTs genes *Dnmt3a* and *Dnmt3l* were significantly upregulated in GOF/GFP^+^ L-ESCs compared with GOF/GFP^−^ cells from the L-ESC (day 5) adaptation process (Figure S3B). Moreover, the transcription levels of genes known to influence DNA methylation levels, such as *Prdm14* and *Nanog*, were significantly downregulated in L-ESCs (Figure S3B).

Notably, many histone genes exhibit lower expression levels in L-ESCs compared with 2i/L-ESCs (Figure S3C). This demonstrates that post-transcriptional regulation and epigenetic modifications may incorporate into L-ESC pluripotency, but the precise regulatory mechanism still needs to be further investigated. Meanwhile, RNA-seq data also indicated that DNA methylation-related genes *Dnmt1*, *Uhrf1*, *Dppa3*, *Zfp57*, and *Trim28* (Greenberg and Bourc'his, 2019; Messerschmidt et al., 2014) were highly expressed in high-passage (p20) L-ESCs compared with 2i/L-ESCs (p21) (Figure S3D). In addition, *Tet1* (ten-eleven translocation 1) and *Tet2* are highly expressed in L-ESCs, and the expression of *Tet3* is low in L-ESCs (Figure S3E). This might relate to promoter demethylation of pluripotency-related genes in L-ESCs. Nevertheless, the regulatory relationship between genome-wide methylation and promoter demethylation of pluripotency genes in L-ESCs needs further investigation.

Next, we examined the dynamic changes in DNMT3A level in the L-ESC adaptation process. Upon withdrawal of PD0325901 and CHIR99021, heterogeneous expression of DNMT3A was detected in nuclei of L-ESCs at day 5, and in long-term culture the DNMT3A protein level was significantly increased in p27 stage L-ESCs (Figure 3B), consistent with the higher methylation in L-ESCs and the notion that PD0325901 promotes downregulation of DNA methylation (Choi et al., 2017; Yagi et al., 2017). The results showed that DNMT3A is an important factor in regulating DNA methylation in L-ESCs, which possess a hypermethylation state.

Proper genomic imprinting is essential for embryonic development (Plasschaert and Bartolomei, 2014; Wu et al., 2014). We further performed genomic imprinting analysis on L-ESCs, S/L-ESCs, and 2i/L-ESCs. Notably, compared with 2i/L-ESCs, the DNA methylation levels at imprinting control regions (ICRs) were markedly higher in L-ESCs and were similar to those in S/L-ESCs (Figure 3D). From these results, we conclude that L-ESCs exhibit global genomic hypermethylation and reserve genomic methylation in the majority of ICRs.

### Serum Treatment Increases the Efficiency of LIF-Dependent ESC Adaptation

**Figure 4:**
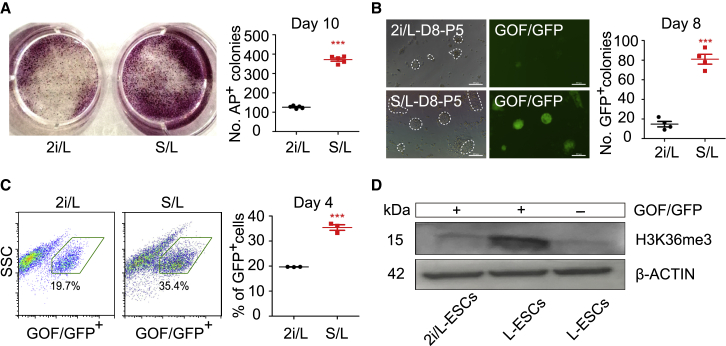
Serum Improves the Efficiency of L-ESC Adaptation (A) Left: alkaline phosphatase (AP) staining on 2i/L-ESCs and S/L-ESCs (2i/L-ESCs cultured in S/L medium for 5 days) that were switched to L medium for 10 days of culture. Right: quantification of AP-positive colonies after 10 days of culture. Error bars are mean ± SD (n = 5). The p values were calculated by two-tailed Student's *t* test, ^∗∗∗^p < 0.05. (B) Left: GOF/GFP^+^ colonies on 2i/L-ESCs and S/L-ESCs (2i/L-ESCs cultured in S/L medium for 5 days) that were switched to L medium for 8 days of culture. Scale bars, 100 μm. Right: quantification of GOF/GFP^+^ colonies after 8 days of culture. Error bars are mean ± SD (n = 4). The p values were calculated by two-tailed Student's *t* test, ^∗∗∗^p < 0.05. (C) Left: FACS based on GOF/GFP^+^ cells, after 2i/L-ESCs and S/L-ESCs (2i/L-ESCs cultured in S/L medium for 5 days) were switched to L medium for 4 days of culture. Right: percentage of GOF/GFP^+^ cells after 4 days of culture. Error bars are mean ± SD (n = 3). The p values were calculated by two-tailed Student's *t* test, ^∗∗∗^p < 0.05. (D) Western blotting analysis for H3K36me3 in early adaptation stage (day 5) GOF/GFP^+^ and GOF/GFP^−^ L-ESCs (results of three independent experiments).

Because S/L-ESCs possess high levels of DNA methylation (Habibi et al., 2013), we next asked if serum treatment (prior to adaptation of L-ESCs) may enhance the DNA methylation and then increase the efficiency of LIF-dependent ESC adaptation. We switched 2i/L-ESCs to S/L medium for 5 days and then cultured induced S/L-ESCs in L medium to assess the LIF-dependent ESC adaptation efficiency. Our result indicates that S/L induction for 5 days significantly increased the number of AP^+^ colonies compared with 2i/L-ESCs (Figures 4A and S4A). Consistent with this, 5-day S/L-induced 2i/L-ESCs were seeded into 24 wells in L medium, and the number of GOF/GFP^+^ colonies obtained from the S/L induction group was drastically increased compared with 2i/L-ESCs (Figure 4B). To confirm this, flow cytometry analysis showed that the percentage of GOF/GFP^+^ cells in the S/L induction group was also increased compared with 2i/L-ESCs (Figure 4C). Furthermore, we tested this adaptation process of advanced stem cells (ASCs) (Bao et al., 2018; Wu et al., 2020) in LIF-alone medium and showed that ASCs can also be efficiently adapted into LIF-dependent ESCs using L medium (Figures S4B and S4C).

### DNMTs Play an Important Role in L-ESC Self-Renewal

Next, we asked whether DNMTs are critical for this adaptation, and investigated the role of DNMTs in this early adaptation process. We separated GOF/GFP^+^ and GOF/GFP^−^ L-ESCs from early adaptation processes (day 5) by FACS. As expected, *Dnmt3a* and *Dnmt3l* expression levels in GOF/GFP^+^ L-ESCs were significantly higher than in GOF/GFP^−^ L-ESCs (Figure S3B), as well as DNMT3A protein level (Figure 3C). Interestingly, we also found a higher expression level of H3K36me3 in GOF/GFP^+^ early adaptation stage (day 5) L-ESCs (Figure 4D). This result is consistent with recent reports of H3K36me3 as a guard for the DNA methylation process (Xu et al., 2019).

**Figure 5:**
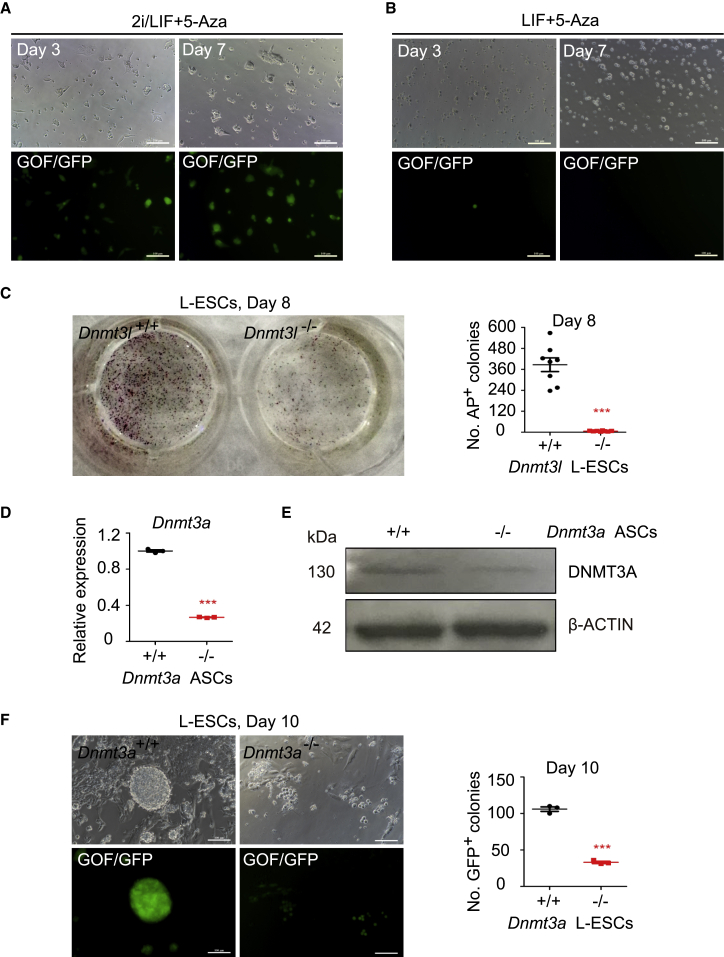
DNMTs Play an Important Role in L-ESC Self-Renewal (A) 2i/L-ESCs were treated with 5-Aza for 3 and 7 days; 2i/L-ESCs retained their typical dome-shaped clonal morphology (results of three independent experiments). Scale bars, 100 μm. (B) L-ESCs were treated with 5-Aza for 3 and 7 days; a few GOF/GFP^+^ L-ESCs survived after 7 days of 5-Aza treatment and finally underwent apoptosis (results of three independent experiments). Scale bars, 100 μm. (C) Left: AP staining on wild-type ESCs and *Dnmt3l*^−/−^ ESCs switched to L medium for 8 days of culture. Right: quantification of AP^+^ colonies after 8 days of culture. Error bars are mean ± SD (n = 8). The p values were calculated by two-tailed Student's *t* test, ^∗∗∗^p < 0.05. (D) Relative expression of *Dnmt3a* by qPCR in *Dnmt3a*^−/−^ ASCs and *Dnmt3a*^+/+^ ASCs. Error bars are mean ± SD (n = 3). The p values were calculated by two-tailed Student's *t* test, ^∗∗∗^p < 0.05. (E) Western blotting analysis for DNMT3A in *Dnmt3a*^−/−^ ASCs and *Dnmt3a*^+/+^ ASCs (results of three independent experiments). (F) Left: GOF/GFP^+^ colonies on wild-type ASCs and *Dnmt3a*^−/−^ ASCs switched to L medium for 10 days of culture. Right: quantification of GOF/GFP^+^ colonies after 10 days of culture. Error bars are mean ± SD (n = 8). The p values were calculated by two-tailed Student's *t* test, ^∗∗∗^p < 0.05.

We next examined the role of DNMTs in regulating L-ESC self-renewal processes using the DNMT inhibitor 5-aza-2′-deoxycytidine (5-Aza). 5-Aza has been widely used as a DNMT inhibitor to experimentally induce gene expression and cellular differentiation (Beyrouthy et al., 2009; Juttermann et al., 1994). We cultured 2i/L-ESCs and L-ESCs in their respective media with 5-Aza and observed morphological changes in both 2i/L-ESCs and L-ESCS. 5-Aza-treated 2i/L-ESCs retained their typical dome-shaped clonal morphology and were able to stably propagate for at least 10 passages (Figures 5A and S5A). In addition, there were slight changes in the expression levels of pluripotent genes (including *Nanog*, *Sox2*, and *Prdm14*) between 5-Aza-treated 2i/L-ESCs and untreated 2i/L-ESCs (Figure S5B). However, L-ESCs treated with 5-Aza failed to maintain self-renewal. There were few GOF/GFP^+^ L-ESCs that survived after 7 days of 5-Aza treatment and the cells underwent apoptosis eventually (Figure 5B). In addition, we performed western blotting analysis on 48-h 5-Aza-treated L-ESCs and 2i/L-ESCs compared with control L-ESCs and 2i/L-ESCs. The results show that 5-Aza treatment significantly reduces DNMT3A, DNMT3B, DNMT3L, and DNMT1 protein levels in L-ESCs and 2i/L-ESCs (Figure S5C). The above results show that 5-Aza treatment affects self-renewal of L-ESCs mainly through DNMTs.

To further investigate the important role of DNMTs in LIF-dependent ESC adaptation processes, we used *Dnmt3l* knockout ESCs (*Dnmt3l*^−/−^ ESCs), which were cultured in S/L medium (Figure 5C), and a *Dnmt3a* knockout ASC line (*Dnmt3a*^−/−^ ASCs), which was generated in ABC/L medium (N2B27 basic medium supplemented with activin A, BMP4, CHIR99021, and LIF) (Figures 5D and 5E) (Bao et al., 2018; Wu et al., 2020) and switched to chemically defined L medium. As expected, both *Dnmt3l* and *Dnmt3a* knockout ESCs showed significantly reduced efficiency of LIF-dependent ESC adaptation (Figures 5C and 5F). Whereas wild-type ESCs and ASC-derived L-ESCs displayed normal self-renewal and proliferation, the proliferation of *Dnmt3l*^−/−^ and *Dnmt3a*^−/−^ L-ESCs decreased dramatically (Figures 5C and 5F). In addition, to avoid influence from the original culture system, we used the same approach and adapted *Dnmt3l*^−/−^ ESCs (S/L medium) and *Dnmt3a*^−/−^ASCs (ABC/L medium) to 2i/LIF medium for 20 days, and followed by switching to L medium to measure the derivation efficiency of *Dnmt3l*^−/−^ and *Dnmt3a*^−/−^ ESCs. Compared with S/L-cultured *Dnmt3l*^−/−^ ESCs and ABC/L-cultured *Dnmt3a*^−/−^ ASCs, both groups showed significantly reduced L-ESC derivation efficiency (Figure S5D). This result showed that the initial DNA methylation level affects the efficiency of L-ESC derivation. In addition, we found that *Dnmt3a*^−/−^ and *Dnmt3l*^−/−^ cells in L medium could be passaged more than 10 times and remained in the self-renewal and heterogenetic state as assayed three times by FACS (Figure S5E), failing to convert to the homogenetic state like the above L-ESCs. In addition, recent studies have focused on the H3K27me3 state in 2i/L-ESCs and S/L-ESCs (Kumar and Elsasser, 2019; McLaughlin et al., 2019; Shukla et al., 2020; van Mierlo et al., 2019). Notably, we found that the level of H3K27me3 in L-ESCs is higher than in 2i/L-ESCs and S/L-ESCs, which may show that L-ESCs are in a special new state, different from 2i/L-ESCs and S/L-ESCs (Figure S5F). Taken together, our data demonstrate that DNMTs promote the induction of LIF-dependent ESC adaptation.

### *In Vitro* and *In Vivo* Differentiation Ability of L-ESCs

**Figure 6:**
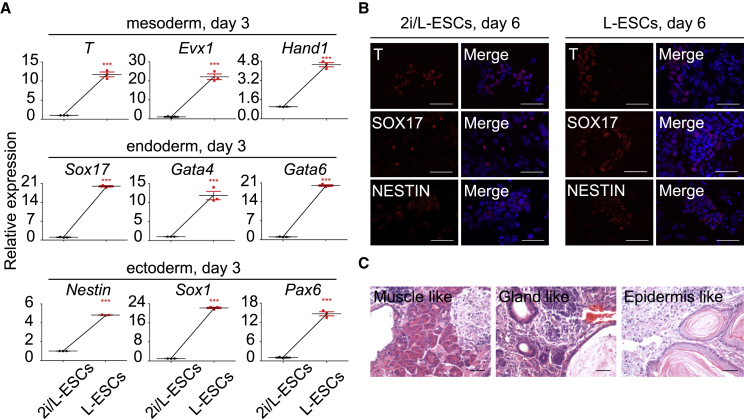
*In Vitro* and *In Vivo* Differentiation Ability of L-ESCs (A) Relative expression of mesoderm, endoderm, and ectoderm genes measured by qPCR, after L-ESCs underwent 3 days of *in vitro* differentiation. Error bars are mean ± SD (n = 3). The p values were calculated by two-tailed Student's *t* test, ^∗∗∗^p < 0.05. (B) Immunostaining of T, SOX17, and NESTIN, after 2i/L-ESCs and L-ESCs underwent 6 days of *in vitro* differentiation (results of three independent experiments). Scale bars, 50 μm. (C) Mature teratomas from L-ESCs. Left: mesoderm, muscle-like cells. Middle: endoderm, gland-like cells. Right: ectoderm, epidermis-like cells. The sections were stained with H&E (results of three independent experiments). Scale bars, 50 μm.

An important criterion for pluripotent ESCs is the ability to differentiate *in vitro* and *in vivo* (De Los Angeles et al., 2015). Upon 2i and LIF withdrawal, pluripotent ESCs differentiate into three germ layers, mesoderm, endoderm, and ectoderm (Shen et al., 2008). We tested the basal expression levels of lineage markers in 2i/L-ESCs and L-ESCs. Our results indicated that several lineage markers were altered, with reduced expression of *Hand1*, *Gata6*, and *Sox17*, and increased expression of *T*, *Sox1*, and *Nestin*, while some were unaltered, such as *Evx1*, *Gata4*, and *Pax6* in 0-day L-ESCs compared with 2i/L-ESCs (Figures S6A). Further to ESC differentiation, we cultured 2i/L-ESCs and L-ESCs in N2B27 basic medium, without 2i/L and LIF. After 3- and 6-day differentiation, we performed qPCR analysis and immunostaining. Interestingly, after 3-day differentiation, the expression levels of all mesoderm, endoderm, and ectoderm genes were significantly increased in L-ESCs compared with 2i/L-ESCs (Figure 6A). Compared with 3-day differentiation, 6-day culturing significantly increased mesoderm, endoderm, and ectoderm gene expression level in 2i/L-ESCs, but found only *T* and *Sox17* increased in L-ESCs after 6-day culture (Figures S6B and S6C). This indicates that L-ESCs have strong flexibility and differentiation ability that depends on environment changes. Nevertheless, 6-day differentiation ability between 2i/L-ESCs and L-ESCs was not significantly different for mesoderm, endoderm, and ectoderm marker gene expression and protein levels (Figures 6B and S6D). In addition, similar to 2i/L-ESCs, L-ESCs also generated teratomas that contained derivatives of the three germ layers (Figure 6C). The results showed that L-ESCs have differentiation ability both *in vitro* and *in vivo* and are able to express important differentiation genes in a shorter space of time compared with 2i/L-ESCs.

### Contribution of L-ESCs to Full-Term Embryonic Development

**Figure 7:**
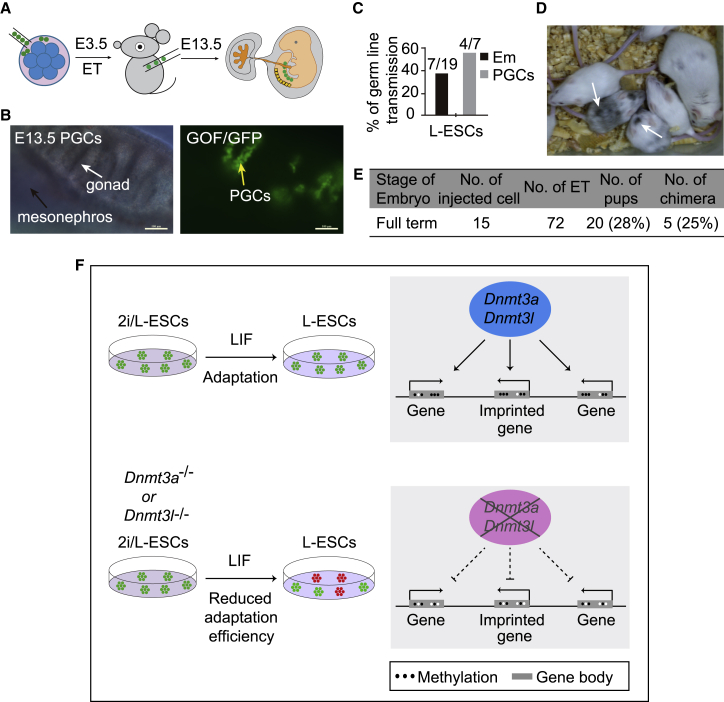
Capability of L-ESCs for Full-Term Embryonic Development (A) Schematic of eight-cell embryo injection protocol. (B) Germline transmission of L-ESCs in E13.5 chimeras. Primordial germ cells (PGCs) are shown by GOF/GFP^+^ cells (arrow). Black arrow, mesonephros; white arrow, gonad; yellow arrow, gonadal PGCs (n = 2 independent experiments). Scale bars, 100 μm. (C) Summary of E13.5 chimera assays by L-ESC injection. The black bar shows the percentage of chimeras among the collected E13.5 conceptuses (Em, embryonic tissues); gray bar shows integration into PGCs among the recovered E13.5 chimeras (n = 2 independent experiments). (D) Chimeric pups generated by injecting L-ESCs into ICR host blastocysts (n = 3 independent experiments). (E) The summary of full-term chimeric pups derived from L-ESCs (n = 3 independent experiments). (F) Schematic of how DNA methylation affects the LIF-dependent ESC adaptation process.

Finally, we tested the *in vivo* developmental potential of L-ESCs in chimeric embryos. Using L-ESCs derived from 2i/L-ESCs, we injected L-ESCs into eight-cell-stage embryos (Figure 7A). We noticed that L-ESCs successfully integrated into E13.5 germlines of chimeras. Notably, 36.8% (7/19) of recovered embryos showed chimeric contribution and 57.1% (4/7) of chimeric embryos displayed germline contribution (Figures 7B and 7C). We further tested whether it is possible to obtain L-ESC-derived postnatal chimeric mice. Of 20 pups born, 5 L-ESC-derived chimeras (25%) were obtained (Figures 7D and 7E). Hence, these data demonstrate the L-ESCs have ESC pluripotency and chimeric competency for both germlines and full-term development.

## Discussion

ESCs are derived from the ICM of the blastocyst and can self-renew indefinitely *in vitro* (Ying et al., 2003, 2008). The signals of WNT, ERK, and JAK/STAT3 are main regulators that combine to control pluripotency; however, the precise functions of the individual signaling pathways are unclear (Ohtsuka et al., 2015). In this study, we present the induction of one new cell type, L-ESCs from 2i/L-ESCs, which depend on JAK/STAT3 signaling alone, and provide new insights into the naive pluripotent stem cells. In particular, L-ESCs show higher DNA methylation levels than 2i/L-ESCs (Figure 3A), and based on transcriptional level, L-ESCs appear to be at an intermediate state between naive ESCs and primed EpiSCs (Figure 2A). We also find that genomic imprints are more stable in L-ESCs relative to 2i/L-ESCs (Figure 3D). Based on the gene expression and DNA methylome analysis, L-ESCs appear to be at an intermediate state between naive ESCs and primed EpiSCs, and may represent stable cells with the characteristics of the early postimplantation epiblast.

LIF signaling includes JAK/STAT, MARK, and PI(3)K pathways, and stimulates a state of self-renewal, as well as determining the fate of cells (Ohtsuka et al., 2015). In mouse ESCs, it is generally believed that LIF signaling is skewed toward survival and self-renewal, whereas activation of canonical WNT signaling and blockade of FGF/ERK inhibits cell differentiation (Ohtsuka et al., 2015). These L-ESCs maintained self-renewal and pluripotency over passage 40. We show that L-ESCs died in 10 days in medium with JAK inhibitor (Figures 1F and 1G). It is clear that LIF is critical to L-ESCs’ self-renewal and to maintain an undifferentiated state. The majority of 2i/L-ESCs undergo differentiation in this regime, and only a small proportion of cells that highly express DNMT3A indicates the presence of naive ESCs, which JAK/STAT3 may favor to bind to cofactors or an intrinsic factor that promotes self-renewal. Recently Tai et al. reported that STAT3 signaling functions in a binary “on/off” manner; however, they used S/L medium, and the defined mechanism needs to be further explored (Tai et al., 2014).

DNA methylation is important for mammalian embryonic development, and DNA-methylation-deficient embryos die at an early stage of development (Greenberg and Bourc'his, 2019). Here, we show that *Dnmt3a* and *Dnmt3l* knockout and 5-Aza treatment affect the efficiency of L-ESC adaptation and L-ESC self-renewal. Interestingly, mouse S/L-ESCs knocked out for *Dnmt1*, *Dnmt3a*, and *Dnmt3b* exhibit DNA hypomethylation, grow stably, and maintain their undifferentiated characteristics (Tsumura et al., 2006). Unlike mouse ESCs, conventional “primed” human ESCs cannot tolerate *Dnmt1* deletion, emphasizing the functional differences between mouse and human ESCs (Liao et al., 2015). Here, we suggest that ESCs cultured in LIF alone exhibit medium-dependent DNA hypermethylation, and *Dnmt3a* or *Dnmt3l* knockout L-ESCs fail to maintain the homogenetic state. Notably, LIF-dependent ESC adaptation efficiency is significantly reduced in *Dnmt3a* or *Dnmt3l* knockout ESCs (Figure 7F). Recently, multiple studies suggested that H3K36me3 participates in cross talk with other chromatin marks and promotes *de novo* DNA methylation by interacting with DNMTs and SETD2 (Xu et al., 2019). H3K36me3 is responsible for establishing and safeguarding the maternal epigenome (Xu et al., 2019). Our results showed that H3K36me3 and DNMT3A were highly expressed in L-ESCs. Epigenetics, including genomic imprinting, has widespread roles in mammals, affecting embryonic and placental development and transmission of nutrients to the fetus, and regulating critical aspects of mammalian physiology, such as metabolism, neuronal development, and adult behavior (Plasschaert and Bartolomei, 2014). We show that L-ESCs reserve hypermethylated imprinting genes, which poses the unique feature of L-ESCs. In conclusion, this study demonstrates that LIF alone is capable of supporting mouse ESC pluripotency, and DNMTs play an important role in L-ESC derivation and self-renewal.

## Experimental Procedures

### Derivation of L-ESCs

2i/L-ESCs (1 × 10^5^) were switched to fibronectin-coated (16.7 μg/mL, Millipore) 24-well cell culture plates containing L medium, which is N2B27 medium supplemented with LIF (1,000 IU/mL, Millipore), and we call these cells L-ESCs. Further information is provided in Supplemental experimental procedures.

### Immunostaining

Cultured ESCs were briefly washed with PBS and fixed in 4% paraformaldehyde in PBS for 15 min at room temperature. Antibody staining was carried out in the same buffer at 4°C overnight and secondary antibody was added for 1 h at room temperature in the dark. Further information is provided in Supplemental experimental procedures.

### Production of Chimeras

Eight to ten ESCs were injected gently into ICR mouse eight-cell-stage embryos using a piezo-assisted micromanipulator attached to an inverted microscope. The injected embryos were cultured in KSOM medium (Millipore) at 37°C in a 5% CO_2_ atmosphere overnight and then transferred to the uteri of pseudopregnant ICR mice at 2.5 days post coitus. The embryos were isolated at embryonic stage E13.5 and checked for germline transmission. Full-term chimeras were confirmed by the coat color pattern of the pups at birth.

### Karyotyping

ESCs were prepared for cytogenetic analysis by treatment with colcemid (Sigma) at a final concentration of 0.1 μg/mL for 3 h to accumulate cells in metaphase. Cells were then exposed to 0.075 M KCl for 25 min at 37°C and fixed with 3:1 methanol:acetic acid. Air-dried slides were generated and G-banded following standard GTG banding protocols.

### Generation of *Dnmt3a* knockout ASC Lines

Guide RNA sequences were cloned into the plasmid px459 (Addgene, 62988). px459 plasmids containing *Dnmt3a* guide RNAs were co-transfected into digested ASCs using Lipofectamine 2000 (Thermo Fisher). Further information is provided in Supplemental experimental procedures.

### RNA Extraction and Sequencing

Total RNA was extracted from approximately 1–2 × 10^6^ cells using an RNeasy Mini Kit (QIAGEN) according to the recommendations of the manufacturer. Further information is provided in Supplemental experimental procedures.

### RNA-seq Data Analysis

Before alignment, raw data were first trimmed to remove reads with more than 10% low-quality bases and to trim adaptors. Then the clean reads were mapped to the mouse reference genome (mm10) with Tophat (2.0.12) with default settings (Trapnell et al., 2009). Further information is provided in Supplemental experimental procedures.

### Genomic DNA Isolation and WGBS Library Preparation

Following the manufacturer's instructions, genomic DNA was extracted from stem cells using the DNeasy Blood & Tissue Kit (Qiagen). Remaining RNA was removed by treating with RNase A. Further information is provided in Supplemental experimental procedures.

### DNA Methylation Analysis

WGBS reads were trimmed with Trim Galore (v.0.3.3) to remove adaptors and low-quality bases. Then we used Bismark (v.0.7.6) (Krueger and Andrews, 2011) to map the clean reads to the mouse reference genome (mm10) with a paired-end and non-directional model; then the unmapped reads were realigned to the same genome with a single-end and non-directional model. Further information is provided in Supplemental experimental procedures.

### Statistical Analysis

All values are depicted as the mean ± SD. Statistical parameters, including statistical analysis, statistical significance, and n value, are reported in the figure legends and supplemental figure legends. Statistical analyses were performed using Prism software (GraphPad Prism v.6). The significance of differences was measured using an unpaired two-tailed Student's *t* test. A value of p < 0.05 was considered significant.

## Accession Numbers

The RNA-seq data are available through the NCBI Sequence Read Archive under the ID PRJNA601004. WGBS data have been deposited in the NCBI Gene Expression Omnibus under accession no. GSE142799. All data that support the conclusions in the study are available from the authors on reasonable request.

## Author Contributions

B.W., F.T., M.A.S., X.L., and S.B. designed the experiments, prepared and approved the manuscript. B.W., Y.L., B.Z., Y.W., and Y.F. conducted the experiments. B.L. analyzed the RNA-seq data. L.L. and J.G. prepared whole-genome bisulfite-sequencing experiments and analyses of bisulfite-sequencing data. C.C. and S.L. helped proof the manuscript.

## Declaration of Interest

The authors declare that they have no competing interests.

## Supplemental Information

Document S1. Supplemental Experimental Procedures, Figures S1–S6, and Tables S1 and S2

Document S2. Article plus Supplemental Information
