# Supplementary material for: DNMTs Play an Important Role in Maintaining the Pluripotency of Leukemia Inhibitory Factor-Dependent Embryonic Stem Cells
Source: Stem Cell Reports. 2021 Feb 25;16(3):582–96. doi: 10.1016/j.stemcr.2021.01.017 (PMC7940253; doi:10.1016/j.stemcr.2021.01.017)
Supplement: Document S1. Supplemental Experimental Procedures, Figures S1–S6, and Tables S1 and S2 [file mmc1.pdf]

Stem Cell Reports, Volume *16*

Supplemental Information

DNMTs Play an Important Role in Maintaining the Pluripotency of Leukemia Inhibitory Factor-Dependent Embryonic Stem Cells

Baojiang Wu, Yunxia Li, Bojiang Li, Baojing Zhang, Yanqiu Wang, Lin Li, Junpeng Gao, Yuting Fu, Shudong Li, Chen Chen, M. Azim Surani, Fuchou Tang, Xihe Li, and Siqin Bao

Stem Cell Reports

Supplemental Information

DNMTs Play an Important Role in Maintaining the Pluripotency of Leukemia Inhibitory Factor Dependent Embryonic Stem Cells

Baojiang Wu, Yunxia Li, Bojiang Li, Baojing Zhang, Yanqiu Wang, Lin Li, Junpeng Gao, Yuting Fu, Shudong Li, Chen Chen, M. Azim Surani, Fuchou Tang, Xihe Li, and Siqin Bao

Inventory of Supplemental Information

Supplemental Figures and Legends

Figure S1. Characteristics of L-ESCs. Related to Figure 1

Figure S2. Analyses of Molecular Features of L-ESCs. Related to Figure 2

Figure S3. Upregulation of DNA Methylation Level in L-ESCs. Related to Figure 3

Figure S4. Serum Improves the Efficiency of L-ESCs Adaptation. Related to Figure 4

Figure S5. DNMTs Play an Important Role in L-ESCs Self-renew. Related to Figure 5

Figure S6. *In vitro* and *in vivo* Differentiation Ability of L-ESCs. Related to Figure 6

Supplemental Spreadsheets Tables

Table S1. WGBS data coverage and conversion efficiency (Related to Figure 3A)

Table S2. RT-qPCR primers and guide RNA sequences

Supplemental Experimental Procedures

Supplemental reference

SUPPLEMENTARY FIGURE LEGENDS AND TABLES

Figure S1. Characteristics of L-ESCs

(A) Summary of L-ESCs derivation from 2i/L-ESCs.

(B) The summary of fluorescence-activated cell sorting (FACS) based on GOF/GFP positive cells in different passages L-ESCs.

(C) Morphology of L-ESCs at p43.

(D) Alkaline phosphatase (AP) staining on L-ESCs (p24) (results of three independent experiments). Scale bars, 100 μm.

(E) Karyotyping of 2i/L-ESCs (P30, n = 50, results of three independent experiments) and L-ESCs (P30, n = 50, results of three independent experiments).

(F) Immunostaining of OCT4, SOX2 and NANOG in 2i/ L-ESCs (results of three independent experiments). Scale bars, 50 μm.

(G) Immunostaining of MERVL and ZSCAN4 in 2i/L-ESCs and L-ESCs (results of three independent experiments). Scale bars, 50 μm.

(H) Immunostaining of H3K27me3 in2i/L-ESCs and L-ESCs (results of three independent experiments). Scale bars, 50 μm.

(I) Derivation of L-ESCs from X/GFP ESCs (results of two independent experiments). Scale bars, 100 μm.

Figure S2. Analyses of Molecular Features of L-ESCs

(A) Heatmap showing general pluripotent markers in L-ESCs (n = 3), 2i/L-ESCs (n = 3), S/L-ESCs (n = 3) and EpiSCs (n = 3) based on RNA-seq data.

(B) Heatmap showing na ïve pluripotent markers in L-ESCs (n = 3), 2i/L-ESCs (n = 3), S/L-ESCs (n = 3) and EpiSCs (n = 3) based on RNA-seq data.

(C) Heatmap showing primed pluripotent markers in L-ESCs (n = 3), 2i/L-ESCs (n = 3), S/L-ESCs (n = 3) and EpiSCs (n = 3) based on RNA-seq data.

(D) Relative expression of WNT signaling related genes by RNA-seq data. Error bars are mean ± SD (n = 3). *P* values were calculated by two tailed Student's *t*-test, $p < 0.05$.

(E) Western blotting analysis for ERK, p-ERK and β-CATENIN in L-ESCs and

2i/L-ESCs (results of three independent experiments).

(F) Heatmap of formative stem cells related genes in L-ESCs (n = 3) and 2i/L-ESCs (n = 3).

Figure S3. Upregulation of DNA Methylation Level in L-ESCs

(A) DNA methylation level at various genomic features in L-ESCs (n = 3), 2i/L-ESCs (n = 3) and S/L-ESCs (n = 2).

(B) Relative expression of *Dnmt3a* and *Dnmt3l* measured by qPCR in GOF/GFP positive and negative L-ESCs; Relative expression of *Prdm14* and *Nanog* measured by qPCR in 2i/L-ESCs, S/L-ESCs and L-ESCs. Error bars are mean ± SD (n = 3). *P* values were calculated by two tailed Student's *t*-test, $p < 0.05$.

(C) Expression levels of all histone genes in L-ESCs (n = 3) and 2i/ L-ESCs (n = 3) based on RNA-seq data.

(D) Heatmap showing DNA maintenance methylation-related genes in 2i/L-ESCs (n = 3) and L-ESCs (n = 3) based on RNA-seq data.

(E) Heatmap showing *Tet1*, *Tet2* and *Tet3* in 2i/L-ESCs (n = 3) and L-ESCs (n = 3) based on RNA-seq data.

Figure S4. Serum Improves the Efficiency of L-ESCs Adaptation

(A) Summary of L-ESCs derivation from 2i/L-ESCs after 5 days S/L medium culture.

(B) Summary of AP positive cloning numbers on 2i/L-ESCs, ASCs and L-ESCs, when 2,000 cells were seeded into 6-well cell culture plate and 6 days culture.

(C) Summary of L-ESCs derivation from ASCs.

Figure S5. DNMTs Play an Important Role in L-ESCs Self-renew

(A) 2i/L-ESCs were treated with 5-Aza after p10, 2i/L-ESCs retained typical dome-shaped clonal morphology (results of three independent experiments). Scale bars, 100 μm.

(B) Relative expression of *Nanog*, *Sox2* and *Prdm14* measured by qPCR in 2i/L-ESCs after 3 days 5-Aza treatment. Error bars are mean ± SD (n = 3). *P* values were

calculated by two tailed Student's *t*-test, $p < 0.05$.

(C) Western blotting analysis for DNMT3A, DNMT3B, DNMT3L and DNMT1 in 2i/L-ESCs, L-ESCs, 5-aza treated 2i/L-ESCs and L-ESCs (results of three independent experiments).

(D) Quantification of colonies number of 2i/L culture different stem cells (*Dnmt3l*-KO ESCs and *Dnmt3a*-KO ASCs) derived L-ESCs. Error bars are mean ± SD (n = 3). *P* values were calculated by two tailed Student's *t*-test, $p < 0.05$.

(E) *Dnmt3a*-KO cells in LIF medium could passage over ten passages, and still kept in self-renewal and heterogenetic state by three times FACS (results of three independent experiments).

(F) Western blotting analysis for H3K27me3 and Histone 3 in 2i/L-ESCs, L-ESCs, 5-aza treated 2i/L-ESCs and L-ESCs and S/L-ESCs (results of three independent experiments).

Figure S6. The Pluripotency of L-ESCs *in vivo* and *in vitro*

(A) Relative expression levels of mesoderm, endoderm and ectoderm related genes in 2i/L-ESCs and L-ESCs before (0-day) *in vitro* differentiation based on RNA-seq data. Error bars are mean ± SD (n = 3). *P* values were calculated by two tailed Student's *t*-test, $p < 0.05$.

(B) Relative expression of mesoderm, endoderm and ectoderm genes measured by qPCR, after 2i/L-ESCs were 3 days and 6 days *in vitro* differentiation. Error bars are mean ± SD (n = 3). *P* values were calculated by two tailed Student's *t*-test, $p < 0.05$.

(C) Relative expression of mesoderm, endoderm and ectoderm genes measured by qPCR, after L-ESCs were 3 days and 6 days *in vitro* differentiation. Error bars are mean ± SD (n = 3). *P* values were calculated by two tailed Student's *t*-test, $p < 0.05$.

(D) Relative expression of mesoderm, endoderm and ectoderm genes measured by qPCR, after 2i/L-ESCs and L-ESCs were 6 days *in vitro* differentiation. Error bars are mean ± SD (n = 3). *P* values were calculated by two tailed Student's *t*-test, p < 0.05.

Figure S1

A

Cell line	Original medium	Derived L-ESCs	Repeated times	Sex
W1	2i/LIF	yes	1	male
W2	2i/LIF	yes	3	male
W4	2i/LIF	yes	2	male
W5	2i/LIF	yes	2	male
W6	2i/LIF	yes	3	male
SQ3.3	2i/LIF	yes	3	female
X/GFP	2i/LIF	yes	2	female
Total	2i/LIF	yes	16	

B

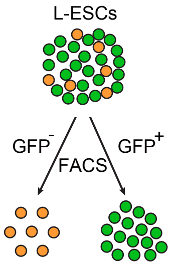

Cell line	medium	No. Sorting	Passage	GFP+ (%)
W2	LIF	1	P23	66
		2	P29	98
		3	P31	99
W6	LIF	1	P14	56
		2	P18	78
		3	P21	91
		4	P33	78
		5	P42	98
W4	LIF	1	P34	97
W5	LIF	1	P34	98
W2	2i/LIF	1	P15	98

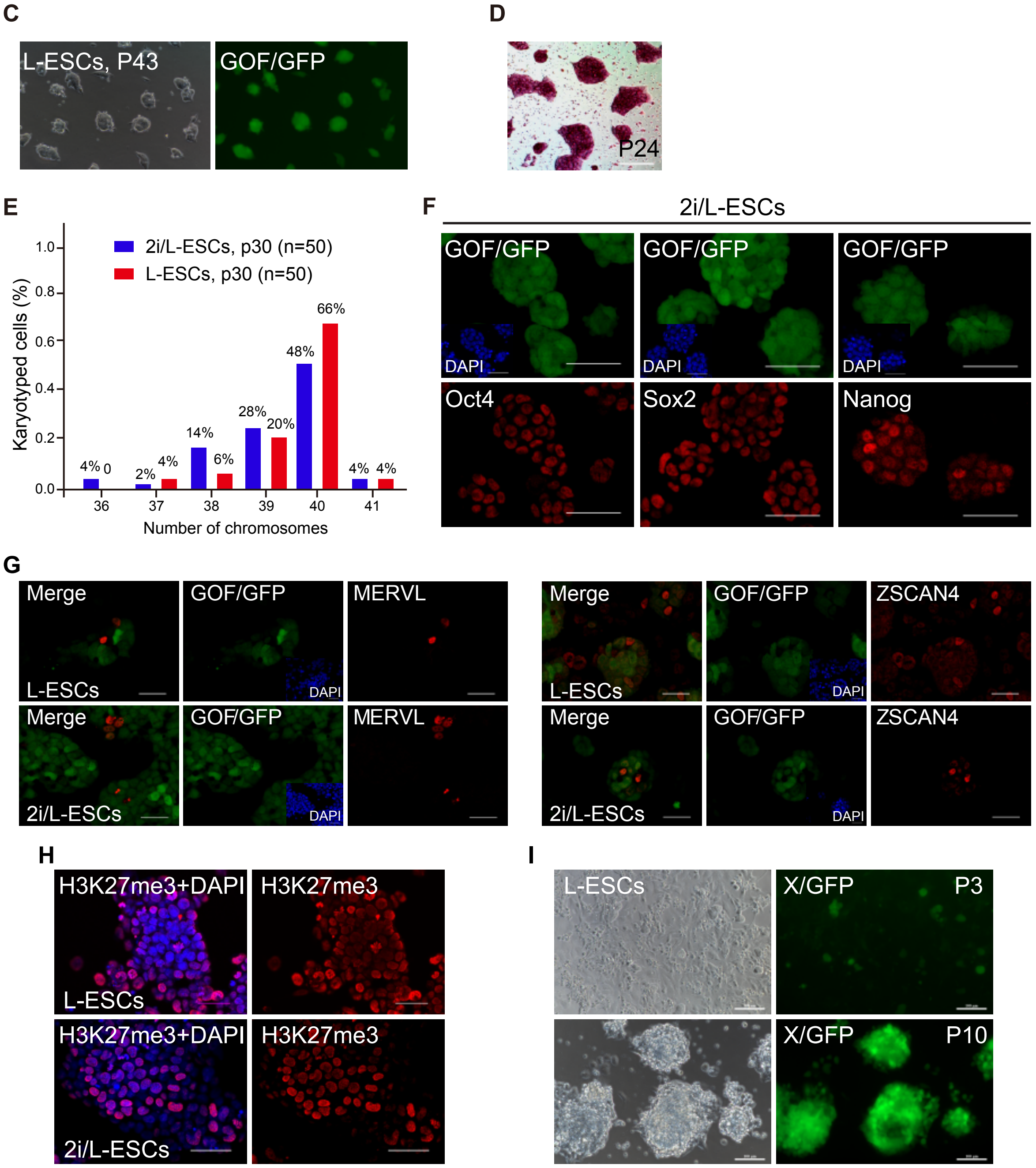

Figure S2

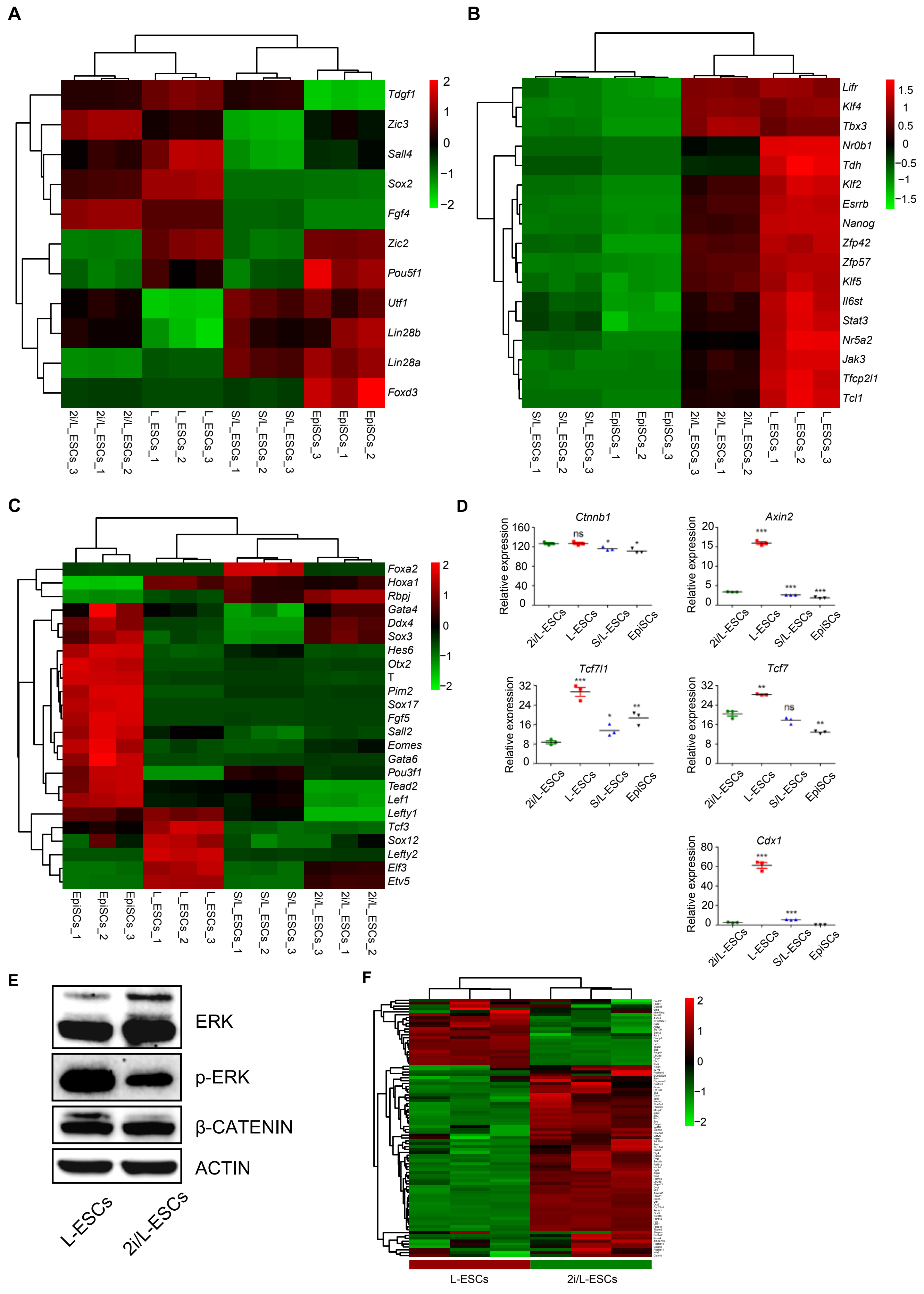
A
Tdgf1
Zic3
Sall4
Sox2
Fgf4
Zic2
Pou5f1
Utf1
Lin28b
Lin28a
Foxd3
2i/L_ESCs_3
2i/L_ESCs_1
2i/L_ESCs_2
L_ESCs_1
L_ESCs_2
L_ESCs_3
S/L_ESCs_1
S/L_ESCs_2
S/L_ESCs_3
EpiSCs_3
EpiSCs_1
EpiSCs_2
B
Lifr
Klf4
Tbx3
Nr0b1
Tdh
Klf2
Esrrb
Nanog
Zfp42
Zfp57
Klf5
Il6st
Stat3
Nr5a2
Jak3
Tfcp2l1
Tcl1
S/L_ESCs_1
S/L_ESCs_2
S/L_ESCs_3
EpiSCs_1
EpiSCs_2
EpiSCs_3
2i/L_ESCs_3
2i/L_ESCs_1
2i/L_ESCs_2
L_ESCs_1
L_ESCs_2
L_ESCs_3
C
Foxa2
Hoxa1
Rbpj
Gata4
Ddx4
Sox3
Hes6
Otx2
T
Pim2
Sox17
Fgf5
Sall2
Eomes
Gata6
Pou3f1
Tead2
Lef1
Lefty1
Tcf3
Sox12
Lefty2
Elf3
Etv5
EpiSCs_1
EpiSCs_2
EpiSCs_3
L_ESCs_1
L_ESCs_2
L_ESCs_3
S/L_ESCs_1
S/L_ESCs_2
S/L_ESCs_3
2i/L_ESCs_3
2i/L_ESCs_1
2i/L_ESCs_2
D
Ctnnb1
Axin2
Tcf7l1
Tcf7
Cdx1
Relative expression
ns
2i/L-ESCs
L-ESCs
S/L-ESCs
EpiSCs
E
ERK
p-ERK
β-CATENIN
ACTIN
L-ESCs
2i/L-ESCs
F
L-ESCs
2i/L-ESCs

Figure S3

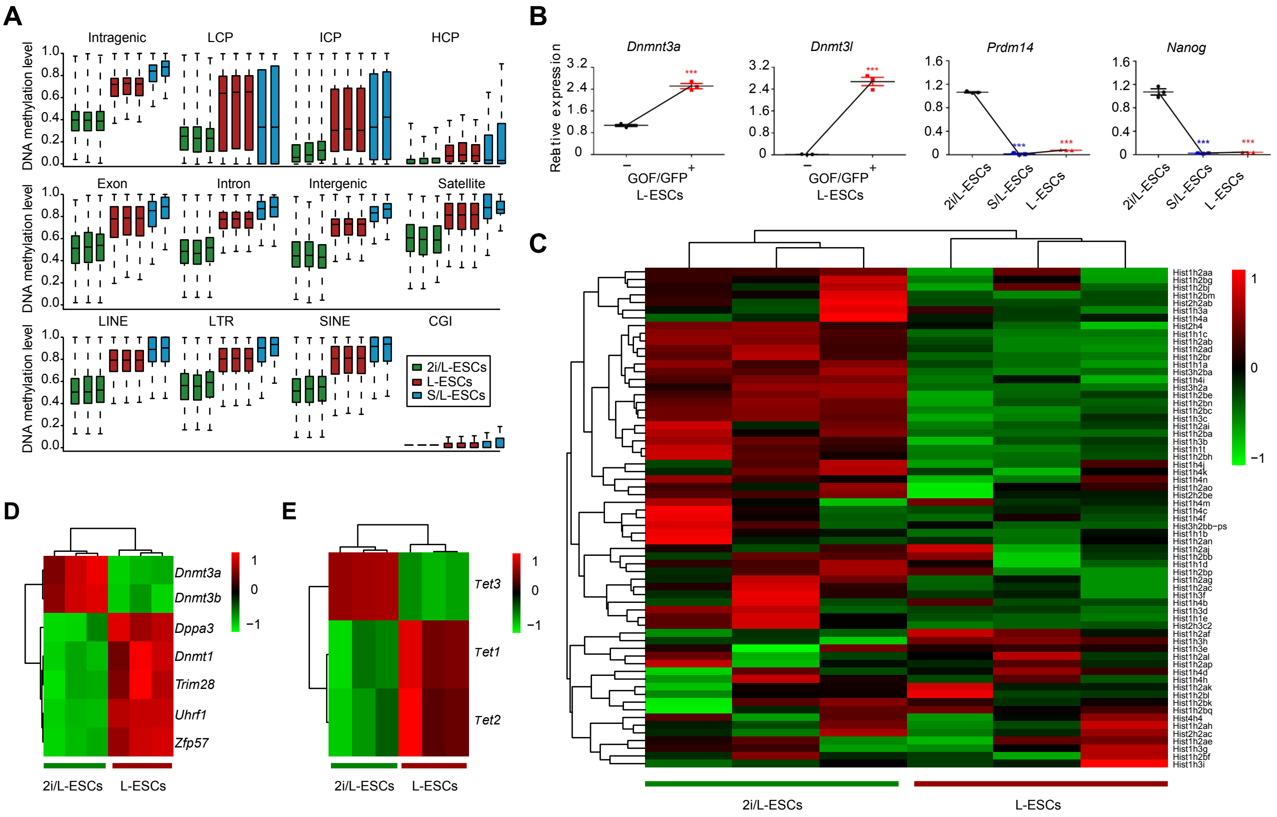
A
Intragenic
LCP
ICP
HCP
Exon
Intron
Intergenic
Satellite
LINE
LTR
SINE
CGI
DNA methylation level
2i/L-ESCs
L-ESCs
S/L-ESCs
B
Relative expression
Dnmnt3a
Dnmt3l
Prdm14
Nanog
GOF/GFP
L-ESCs
2i/L-ESCs
S/L-ESCs
L-ESCs
C
2i/L-ESCs
L-ESCs
D
Dnmt3a
Dnmt3b
Dppa3
Dnmt1
Trim28
Uhrf1
Zfp57
2i/L-ESCs
L-ESCs
E
Tet3
Tet1
Tet2
2i/L-ESCs
L-ESCs

Figure S4

A

Cell line	Original medium	S/L induction and L-ESCs reprogramming	Repeated times
W2	2i/L	yes	3
W6	2i/L	yes	4
W4	2i/L	yes	4
SQ3.3	2i/L	yes	2
J1	S/L	yes	3
Dnmt3l-/-	S/L	yes	3

B

Cell line	W5		329			
Culture medium	2i+LIF	2i+LIF	ABCL	ABCL	LIF	LIF
Number of AP$^+$ colonies	1673	1862	3425	3672	1660	1548

C

Cell line	Original medium	Reprogrammed L-ESCs	Repeated times
G3	ABCL	yes	3
G3-AH	ABCL	yes	2
G12	ABCL	yes	1
329	ABCL	yes	3
Dnmt3a-/-	ABCL	yes	2

Figure S5

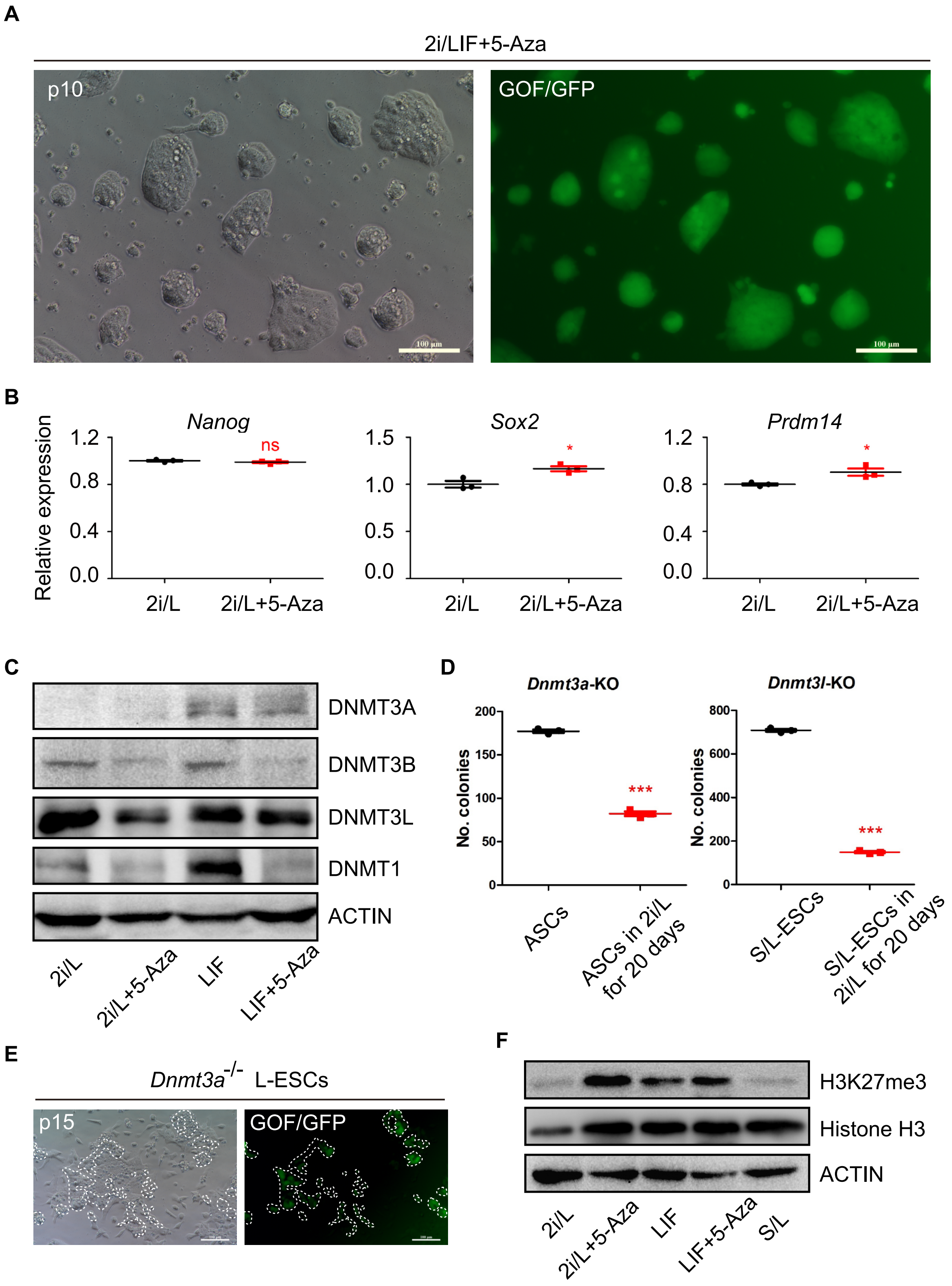
A
2i/LIF+5-Aza
p10
GOF/GFP
B
Relative expression
Nanog
ns
1.2
0.8
0.4
0.0
2i/L
2i/L+5-Aza
Sox2
*
1.5
1.0
0.5
0.0
2i/L
2i/L+5-Aza
Prdm14
*
1.2
0.8
0.4
0.0
2i/L
2i/L+5-Aza
C
DNMT3A
DNMT3B
DNMT3L
DNMT1
ACTIN
2i/L
2i/L+5-Aza
LIF
LIF+5-Aza
D
Dnmt3a-KO
No. colonies
200
150
100
50
0
***
ASCs
ASCs in 2i/L for 20 days
Dnmt3l-KO
No. colonies
800
600
400
200
0
***
S/L-ESCs
S/L-ESCs in 2i/L for 20 days
E
Dnmt3a-/- L-ESCs
p15
GOF/GFP
F
H3K27me3
Histone H3
ACTIN
2i/L
2i/L+5-Aza
LIF
LIF+5-Aza
S/L

Figure S6

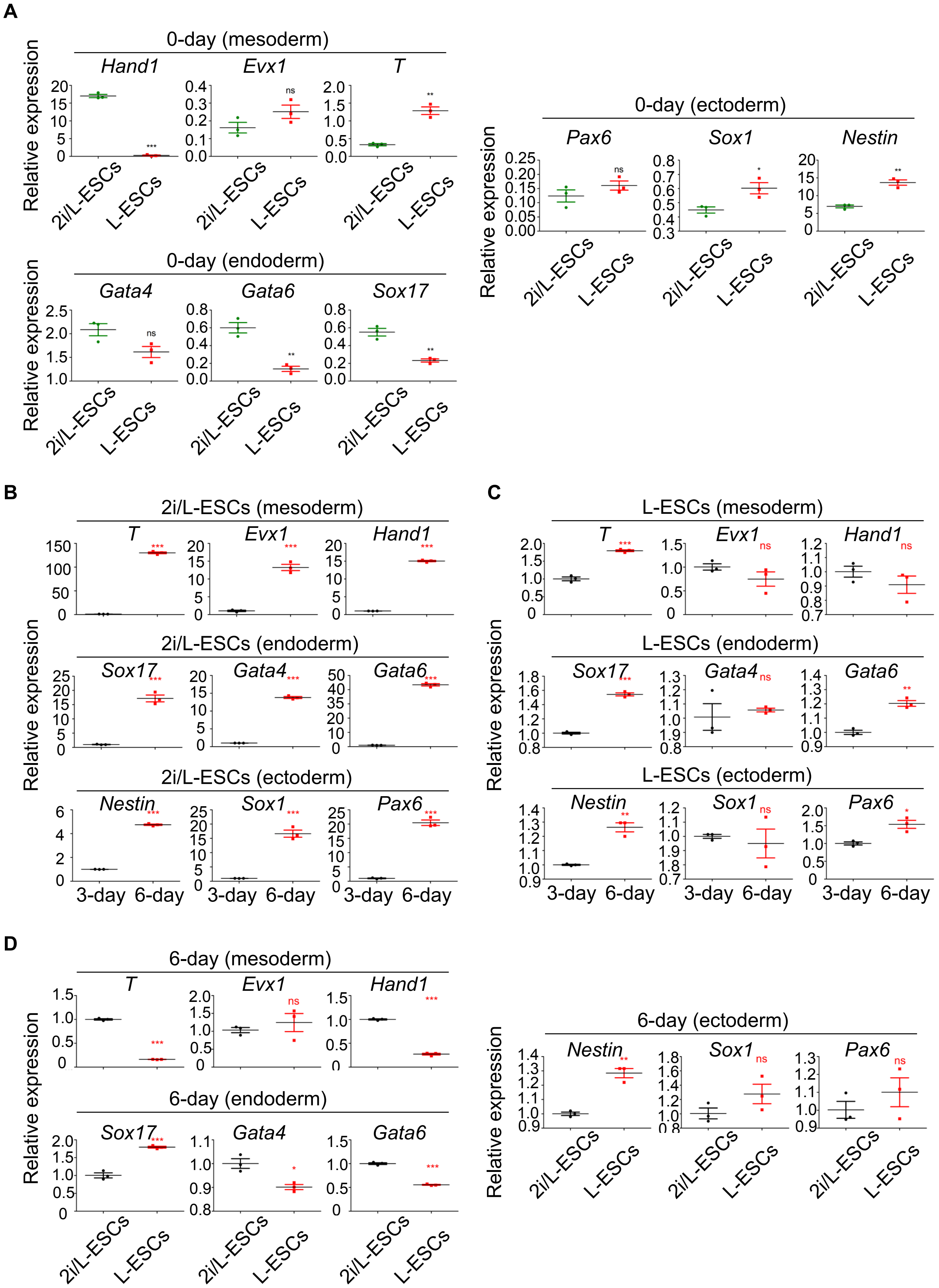
A
0-day (mesoderm)
Hand1
Evx1
T
0-day (endoderm)
Gata4
Gata6
Sox17
0-day (ectoderm)
Pax6
Sox1
Nestin
Relative expression
2i/L-ESCs
L-ESCs
B
2i/L-ESCs (mesoderm)
2i/L-ESCs (endoderm)
2i/L-ESCs (ectoderm)
3-day
6-day
C
L-ESCs (mesoderm)
L-ESCs (endoderm)
L-ESCs (ectoderm)
D
6-day (mesoderm)
6-day (endoderm)
6-day (ectoderm)

Table S1. WGBS data coverage and conversion efficiency (Related to Fig 3A)

Sample	Total sequenced bases(Gb)	Total sequenced reads	Reads after trimming	Unique mapped reads	Mapping efficiency	Coveraged bases (1x)
L-ESCs_rep1	23.36	155,760,956	151,382,966	98,603,675	65.14%	2,317,989,827
L-ESCs_rep2	26.20	174,654,234	168,555,054	106,718,870	63.31%	2,334,743,972
L-ESCs_rep3	24.60	163,969,284	158,835,308	103,120,363	64.92%	2,346,240,160

Sample	Fraction of genome covered	Bisulfite Conversion Rate	No.of unique CpG covered (1x)	No.of unique CpG covered (3x)	No.of unique CpG covered (5x)
L-ESCs_rep1	85.05%	99.96%	30,757,370	14,829,901	5,277,450
L-ESCs_rep2	85.66%	99.96%	30,976,871	15,454,751	5,806,380
L-ESCs_rep3	86.08%	99.96%	31,620,932	15,560,785	5,654,681

Table S2. RT-qPCR Primers and Guide RNA sequences

Gene name	Forward Primer	Reverse Primer
Nanog	CTTTCACCTATTAAGGTGCTTGC	TGGCATCGGTTCATCATGGTAC
Prdm14	CCTGAACAAGCACATGAGA	TGCACTTGAAGGGCTTCTCT
Gata4	TTCCTCTCCCAGGAACATCAAA	GCTGCACAACTGGGCTCTACTT
Gata6	TGCTGGAAATTGCAACAAACC	GTCACGTGGTACAGGCGTCA
Sox17	GTCAACGCCTTCCAAGACTTG	GTAAAGGTGAAAGGCGAGGTG
Brachyury	GAACCTCGGATTCACATCGT	TTCTTTGGCATCAAGGAAGG
Evx1	CCAGTGACCAGATGCGCCGATAC	TCCTTCATGCGCCGGTTCT
Hand1	TCAAAAAGACGGATGGTGGT	GCGCCCTTTAATCCTCTTCT
Dnmt3a	GACTCGCGTGCAATAACCTTAG	GGTCACTTTCCCTCACTCTGG
Dnmt3l	CGGAGCATTGAAGACATC	CATCATCATACAGGAAGAGG
Sox2	GCGGCGGAAAACCAAGA	CCGGGAAGCGTGTACTTATCC
Nestin	CTCGAGCAGGAAGTGGTAGG	TTGGGACCAGGGACTGTTAG
Sox1	GGCCGAGTGGAAGGTCATGT	TCCGGGTGTTCCTTCATGTG
Pax6	GCAGATGCAAAAGTCCAGGTG	CAGGTTGCGAAGAACTCTGTTT
GAPDH	ATGGTGAAGGTCGGTGTGAAC	TCGCTCCTGGAAGATGGTGATG

Guide RNA sequences

Dnmt3a sgRNA1	CACCGCTCATACTCAGGCTCATCGT
Dnmt3a sgRNA1-CS	AAACACGATGAGCCTGAGTATGAGC
Dnmt3a sgRNA2	CACCGGACCCTGCTTCTCCGACTG
Dnmt3a sgRNA2-CS	AAACCAGTCGGAGAAGCAGGGTCC

Genotyping primer

Dnmt3a 89925 Forward Primer	GCCTTGGCTGTGTGAGATTTG
Dnmt3a 90586 Reverse Primer	ATCCTGGAGCCCCAAAGAGC

Supplemental Experimental Procedures

Mice

Animal care and use were conducted in accordance with the guidelines of Inner Mongolia University, China. Mice were housed in a temperature-controlled room with proper darkness-light cycles, fed with a regular diet, and maintained under the care of the Laboratory Animal Unit, Inner Mongolia University, China. The mice were sacrificed by cervical dislocation. This study was specifically approved by the Institutional Animal Care and Use Committee, Inner Mongolia University, China. Oct4-**Δ**PE-GFP (GOF/GFP) transgenic mice (Yoshimizu *et al*, 1999) were used here with a mixed background of MF1, 129/sv, and C57BL/6J strains.

Derivation of 2i/L-ESCs

Mouse embryos blastocysts (E3.5) were isolated from 129/sv females mated with GOF/GFP transgenic males. Green fluorescence indicated that GFP expression of the reporter is under the control of *Oct4* promoter and distal enhancer. This GFP transgene shows expression in the ICM of blastocysts and PGC *in vivo*, and in ESCs (Yoshimizu *et al*, 1999). ESCs culture medium consists of N2B27 medium (Life technology) supplemented with PD0325901 (PD, 1 μM, Miltenyi Biotec), CHIR99021 (CH, 3 μM, Miltenyi Biotec) and leukemia inhibitory factor (LIF, 1000 IU/ml, Millipore), henceforth were called 2i/L medium. Zona pellucida of blastocysts were removed by Acidic Tyrode’s Solution (Sigma-Aldrich), and then placed to 24-well fibronectin-coated (FN, 16.7 μg/ml, Millipore) plate with 2i/L medium. ICM

of blastocysts cultures grew efficiently and formed outgrowing colonies in 5-7 days culture. The resulting colonies were further cutting into smaller pieces by glass needles after 5-7 days culture, and then the colonies passaged by Accutase (Life technology) regularly on at every 2 days interval.

Derivation of L-ESCs

1×10^5 2i/L-ESCs were switched on fibronectin-coated (16.7 μg/ml, Millipore) 24-well cell culture plate containing L-medium which are N2B27 medium supplemented with leukemia inhibitory factor (1000 IU/ml, Millipore), and we call these cells as L-ESCs. Dependent on cell growth, L-ESCs were passage every other day in the early stage. After being cultured for different passages, GOF/GFP positive and negative L-ESCs were purified by flow-cytometry sorting by BD FACSAria (BD Biosciences) and further analysis. GOF/GFP positive purified L-ESCs were passage every other day treated with Accutase (Life technology). L-ESCs were capable of self-renewal for over 40 passages. For inhibitor treatment experiment, we added JAK inhibitor I (0.6 μM, Calbiochem) or 5-Aza (6 μM, Sigma) into L-ESCs culture medium.

Derivation of S/L-ESCs

2i/L-ESCs were switch to fibronectin-coated plate with standard ES medium (Knockout DMEM; Knockout Dulbecco's modified Eagle's medium) supplemented with 20% fetal calf serum, 0.1 mM 2-mercaptoethanol, 2 mM L-glutamine, 0.1 mM

non-essential amino acid, 50 U/ml Penicillin/Streptomycin and 1000U/ml LIF without feeder cells, we named these cells as S/L-ESCs.

Flow Cytometry

GOF/GFP ESCs were harvested by Accutaes and sorting by BD LSRFortessa. Green fluorescence indicated that GFP expression of the reporter is under the control of Oct4 promoter and distal enhancer. This GFP transgene shows expression in the ICM of blastocysts and PGCs *in vivo*, and in ESCs. No GOF/GFP ESCs were used for FACS gating negative control. Measure fluorescence (detector 488 nm channel for GFP) by flow cytometer. Gating out of residual cell debris and measure diploid and tetraploid DNA peaks. A region representing GFP-positive cells were used to identify living cells and collected.

Alkaline Phosphatase (AP) Staining

AP staining was carried out using AP staining kit from Sigma (86R-1KT) according to manufacturer's instructions. Briefly, the cells were fixed by 4% paraformaldehyde for 10 min, and then were stained by AP staining solution for overnight at room temperature.

Cell Differentiation

2i/L-ESCs and L-ESCs were cultured in N2B27 medium for 3 to 6 days withdrawal of PD0325901, CHIR99021 and LIF, and LIF respectively.

Colony Formation Assay

Single 2i/L-ESCs and L-ESCs were seeded at a fibronectin-coated 96-well plates using mouth pipette, containing 2i/L and L-medium, respectively. The cells were cultured for 10 days and the number of colonies was assessed.

Generation of *Dnmt3a* Knockout ASCs Lines

Guide RNA sequences were cloned into the plasmid px459 (Addgene, 62988). px459 containing *Dnmt3a* gRNAs were co-transfected into digested ASCs by Lipofectamine 2000 (Thermo Fisher). Single cell derived colonies were picked and expanded individually in ABC/L (N2B27 basic medium added with Activin A, BMP4, CHIR99021 and LIF) medium with puromycin. Genomic DNA of colonies was extracted using the DNeasy Blood & Tissue Kit, which was further analyzed by genomic PCR. Colonies with the deletion of *Dnmt3a* locus were identified. *Dnmt3a* knockout ASCs (*Dnmt3a*$^{-/-}$ ASCs) were cultured in ABC/L medium without puromycin. Guide RNA sequences and genotyping primer sequences used are given in Table S2.

Teratomas Formation

The 2i/L-ESCs and L-ESCs were disaggregated using Accutase, and 1×10^6 cells were injected into under epithelium of NOD–SCID mice. Three to five weeks after transplantation, tumor(s) were collected and fixed with 4% paraformaldehyde, and

processed for paraffin sectioning. Sections were observed following Hematoxylinand Eosin staining.

Immunostaining

Cultured ESCs were briefly washed with PBS and fixed in 4% paraformaldehyde in PBS for 15 min at room temperature. Cells were permeabilized for 30 min with 1% BSA and 0.1% Triton X-100 in PBS. Antibody staining was carried out in the same buffer at 4℃ for overnight. The slides were subsequently washed three times in 1% BSA, 0.1% Triton X-100 in PBS (5 min each wash), were incubated with secondary antibody for 1h at room temperature in the dark, washed once for 5 min in 1% BSA, 0.1% Triton X-100 in PBS and twice for 5 min in PBS. The slides were then mounted in Vectashield with DAPI (Vector Laboratories) and imaged using a Olympus FV1000 confocal microscope. Primary antibodies used were: anti-OCT4 (BD Biosciences, Catalog Number: 611203, 1:200), anti-NANOG (eBioscience, Catalog Number: 14-5761, 1:500), anti-SOX2 (Santa cruz, Catalog Number: sc-17320, 1:200), anti-H3K27me3 (Upstate, Catalog Number: 07-449, 1:500), anti-ZSCAN4 (Abcam, Catalog Number: ab106646, 1:200), anti-MERVL (HuaAn Bio, Catalog Number: ER50102, 1:100), anti-DNMT3A (abcam, Catalog Number: ab79822, 1:500), anti-NESTIN (BOSTER Bio, Catalog Number: BM4494, 1:50), anti-BRACHYURY (R＆D Systems, Catalog Number: AF2085, 1:100) and anti-SOX17 (R＆D Systems, Catalog Number: AF1924, 1:100). All secondary antibodies used were Alexa Fluor highly crossed adsorbed (Molecular Probes).

Western Blot

Cells were collected with Accutase (Life technology), washed three times with DPBS, and lysed in buffer that contained 20 mM Tris (pH 8.0), 137 mM NaCl, 100 g/l glycerol, 50 g/l Triton X-100, and 4 g/l EDTA; 1 μl PMSF (0.1 M) and 10 μl phosphatase inhibitor (10 g/l) were added per 1 ml lysis buffer immediately before use. Proteins were denatured with 2 × SDS at 95 °C for 5 min. A total of 20 μg denatured protein was run on 8% or 10% SDS–PAGE gel and transferred to polyvinylidene difluoride (PVDF) membrane. Membranes were blocked with 5% nonfat milk in 1 × TBS with 0.05% Tween-20 (TBST) for 1h. Samples were probed with primary antibodies overnight at 4 ℃. The primary antibodies used were anti-DNMT3A (CST, 3598S; dilution 1:1,000), anti-DNMT3B (Abcam, ab78922; dilution 1:2,000), anti-DNMT3L (Abcam, ab3493; dilution 1:2,500), anti-DNMT1 (Abcam, ab19905; dilution 1:1,000), anti-H3K36me3 (Abcam, ab9050; working concentration, 1 μg/ml), anti-phospho-p44/p42 MAPK (p-ERK1/2) (Cell Signaling Technology, 4370; dilution 1:2,000), anti-p44/p42 MAPK (ERK1/2) (Cell Signaling Technology, 4695; dilution 1:1,000), anti- β-CATENIN (Cell Signaling Technology, 8480; dilution 1:1,000) and anti-β-ACTIN (Abcam, ab8227; dilution 1:5,000). Blots were rinsed with TBST. Membranes were incubated with HRP-conjugated secondary antibodies for 60 min at room temperature, and proteins were detected by ECL plus reagent. After rinsing with TBST, Clarity™ Western ECL Substrate (BIO-RAD) was used for visualization, and ChemiDoc™ MP Imaging System (BIO-RAD) was used for band detection.

Real-Time PCR

Total RNA was isolated with the RNeasy Plus Mini Kit (Qiagen) and reverse transcribed into cDNA using the Reverse Transcription System (Promega) according to the manufacturer's instructions. Quantitative real-time PCR (qRT-PCR) was conducted using a LightCycler® 96 Instrument (Roche Molecular Systems) and qRT-PCR reaction was performed with KAPA SYBR FAST qPCR kit (KAPA Biosystems). At least triplicate samples were assessed for each gene of interest, and GAPDH was used as a control gene. Relative expression levels were determined by the $2^{-\Delta\Delta Ct}$ method. Primer sequences used are given in Table S2.

RNA Extraction and Sequencing

Total RNA were extracted from approximately one million to two million cells using RNeasy Mini Kit (QIAGEN) according to the recommendation of manufacturer and then NEBNext® Poly (A) mRNA Magnetic Isolation Module was used to isolate mRNA from total RNA. Using mRNA as input, the first and second strand cDNAs were synthesized by NEBNext® RNA First Strand Synthesis Module and NEBNext® Ultra II Non-Directional RNA Second Strand Synthesis Module, respectively. Final libraries were prepared using KAPA Hyper Prep Kits (8 PCR cycles) and sequenced on HiSeq4000 platform.

RNA-seq Data Analysis

Before alignment, raw data were first trimmed to remove reads with more than 10% low quality bases and to trim adaptors. Then the clean reads were mapped to mouse reference genome (mm10) with Tophat (2.0.12) with default settings (Trapnell *et al*, 2009). HTSeq (0.6.1) was used to do the reads counting, and then RefSeq gene expression level was estimated by RPKM method (Reads per kilobase transcriptome per million reads). Data of RNA-seq of S/L-ESCs and EpiSCs (GSE119985) were downloaded from previous study (Wu *et al*, 2020). *In vivo* data of mouse embryos E2.5-E5.5 were downloaded from ArrayExpress (E-MTAB-2958) (Boroviak *et al*, 2015). Differentially expressed genes (DEGs) in different samples were determined by edgeR package with fold-change ≥ 2 and p value ≤ 0.5 (Robinson *et al*, 2010). Unsupervised hierarchical clustering (UHC) analysis was performed by the R hclust function. Heatmaps of select genes were performed using R heatmap.2 function. Principal component analysis (PCA) analysis was performed with the R prcomp function. Gene ontology analysis was performed using Metascape (http://metascape.org). Trend analysis of DEGs was performed using Short Time-series Expression Miner (STEM) software (Ernst & Bar-Joseph, 2006).

Genomic DNA Isolation and WGBS Library Preparation

Following the manufacturer's instructions, genomic DNA was extracted from stem cells using the DNeasy Blood & Tissue Kit (Qiagen). Remaining RNA was removed by treating with RNase A. Three replicated samples from each of these stem cells

were used for library preparation to ensure repeatability of experiment. In short, 2 μg of genomic DNA spiked with 10 ng of lambda DNA were fragmented to about 300 bp with Covaris S220. Next, end repair and A-ligation were performed to the DNA fragments. Methylated Adaptor (NEB) was then ligated to the DNA fragments. In order to reach >99% bisulfite conversion, the adaptor-ligated DNA was treated twice using EZ-96 DNA Methylation-Direct™ MagPrep (Zymo Research). The resulting single-strand DNA fragments were amplified by 4 PCR cycles using the KAPA HiFi HotStart Uracil+ ReadyMix (2×). At last, the libraries were sequenced on HiSeq4000 platform to generate 150-bp paired-end reads.

DNA Methylation Analysis

Whole genome bisulfite sequencing reads were trimmed with Trim Galore (v0.3.3) to remove adaptors and low quality bases. Then we used Bismark (v0.7.6) (Krueger & Andrews, 2011) to map the clean reads to mouse reference genome (mm10) with a paired-end and non-directional model, then the unmapped reads were realigned to the same genome with a single-end and non-directional model. PCR duplications were removed with command 'samtools rmdup' (v0.1.18). WGBS data of 2i/L-ESCs (GSE119985) (Wu *et al*, 2020) and S/L-ESCs (GSE98517) (Hackett *et al*, 2017) were downloaded from previous study and identically processed. The global DNA methylation level, estimated using a 2 kb window across the genome, and DNA methylation level in each genomic regions was estimated based on 3x CpG sites (CpGs covered more than 3 times). Only regions with more than 3 CpGs covered

were retained. Genomic annotation, like exons, introns and repeat regions were downloaded from UCSC genome browser. Promoters were regions 1 kb upstream and 0.5 kb downstream of transcription start sites (TSS). Imprint control regions (ICR) were obtained from previous study (Xie *et al*, 2012), for the low coverage of published S/L-ESCs data, DNA methylation level on ICRs were estimated based on 1x CpG sites. Locations of ICRs were converted with UCSC LiftOver from mm9 to mm10.

Supplemental Reference

Boroviak, T., Loos, R., Lombard, P., Okahara, J., Behr, R., Sasaki, E., Nichols, J., Smith, A., and Bertone, P. (2015). Lineage-Specific Profiling Delineates the Emergence and Progression of Naive Pluripotency in Mammalian Embryogenesis. Dev Cell *35*: 366-382.

Ernst, J., and Bar-Joseph, Z. (2006). STEM: a tool for the analysis of short time series gene expression data. BMC Bioinformatics *7*: 191.

Hackett, J.A., Kobayashi, T., Dietmann, S., and Surani, M.A. (2017). Activation of Lineage Regulators and Transposable Elements across a Pluripotent Spectrum. Stem Cell Reports *8*: 1645-1658.

Krueger, F., and Andrews, S.R. (2011). Bismark: a flexible aligner and methylation caller for Bisulfite-Seq applications. Bioinformatics *27*: 1571-1572.

Robinson, M.D., McCarthy, D.J., and Smyth, G.K. (2010). edgeR: a Bioconductor package for differential expression analysis of digital gene expression data.

Bioinformatics *26*: 139-140.

Trapnell, C., Pachter, L., and Salzberg, S.L. (2009). TopHat: discovering splice junctions with RNA-Seq. Bioinformatics *25*: 1105-1111.

Wu, B., Li, L., Li, B., Gao, J., Chen, Y., Wei, M., Yang, Z., Zhang, B., Li, S., Li, K. *et al* (2020). Activin A and BMP4 Signaling Expands Potency of Mouse Embryonic Stem Cells in Serum-Free Media. Stem Cell Reports *14*: 241-255.

Xie, W., Barr, C.L., Kim, A., Yue, F., Lee, A.Y., Eubanks, J., Dempster, E.L., and Ren, B. (2012). Base-resolution analyses of sequence and parent-of-origin dependent DNA methylation in the mouse genome. Cell *148*: 816-831.

Yoshimizu, T., Sugiyama, N., De Felice, M., Yeom, Y.I., Ohbo, K., Masuko, K., Obinata, M., Abe, K., Scholer, H.R., and Matsui, Y. (1999). Germline-specific expression of the Oct-4/green fluorescent protein (GFP) transgene in mice. Dev Growth Differ *41*: 675-684.
